# Statistical inference for a constant-stress partially accelerated life tests based on progressively hybrid censored samples from inverted Kumaraswamy distribution

**DOI:** 10.1371/journal.pone.0272378

**Published:** 2022-08-01

**Authors:** Manal M. Yousef, Salem A. Alyami, Atef F. Hashem

**Affiliations:** 1 Department of Mathematics, Faculty of Science, New Valley University, EL-Khargah, Egypt; 2 Department of Mathematics and Statistics, College of Science, Imam Mohammad Ibn Saud Islamic University (IMSIU), Riyadh, Saudi Arabia; 3 Mathematics and Computer Science Department, Faculty of Science, Beni-Suef University, Beni-Suef, Egypt; Universidad Rey Juan Carlos, SPAIN

## Abstract

In this article, we investigate the problem of point and interval estimations under constant-stress partially accelerated life tests. The lifetime of items under use condition is assumed to follow the two-parameter inverted Kumaraswamy distribution. Based on Type-I progressively hybrid censored samples, the maximum likelihood and Bayesian methods are applied to estimate the model parameters as well as the acceleration factor. Under linear exponential, general entropy and squared error loss functions, Bayesian method outcomes are obtained. In addition, interval estimation is achieved by finding approximately confidence intervals for the parameters, as well as credible intervals. To investigate the accuracy of the obtained estimates and to compare the performance of confidence intervals, a Monte Carlo simulation is developed. Finally, a set of real data is analyzed to demonstrate the estimation procedures.

## 1 Introduction

In some situations, due to the continuous progress in manufacturing technology, modern products are designed and built to operate without failure for a long time under standard operating conditions, and then it is very difficult, if not impossible, to obtain failure data for these products. Because of this difficulty, reliability professionals have attempted to devise techniques to quickly induce failures by subjecting the highly reliable products to harsher environmental conditions without adding additional failure modes other than those found under standard operating conditions. To evaluate the life characteristics and reliability performance of such products under standard operating conditions, accelerated life tests (ALTs) are used to quickly obtain information on such products by subjecting them to severe conditions to obtain the required failure data in a short time. The severe conditions may include subjecting the test products to higher voltage, temperature, pressure, vibrations, or combining some of them.

In ALT, there exists either a known acceleration factor or a mathematical model to define the relationship between stress and lifetime. There are, however, certain cases in which neither acceleration factor is known nor such a model exists or is very difficult to assume. To overcome this problem, the partially ALT (PALT) is a strong candidate to perform the life test in such situations. It is a better test to use when testing items under both normal and higher-than-normal stress conditions. Furthermore, it is the most important tool for determining the acceleration factor and, as a result, extrapolating the accelerated test data to the use condition. The PALT incorporates both accelerated and normal life tests. As a result, PALT is appropriate for estimating the acceleration factor.

Various types of stress loading may be applied when performing PALT. One way of applying stress to the test products is the constant-stress test. According to Nelson [1], constant-stress testing has several advantages. For starters, it is easier to maintain a constant-stress level in most tests. Second, for some materials and products, accelerated test models for constant stress are better developed. Third, data analysis for estimating reliability is well developed. In constant-stress PALT, the total test products are first divided into two groups. Products of group 1 are assigned to the standard condition while those of group 2 are allocated to the accelerated condition. Each test product will run at a constant stress level until the product fails or the test is terminated under a certain censorship scheme. Another way of applying stress to the test products is through a step-stress scheme. In step-stress PALT, the test products are initially operated in standard use conditions and, if they do not fail for a predetermined time, then they are run in accelerated conditions until failure occurs or the products are censored, see, for example, [2–5].

For a brief review on constant-stress ALT, AL-Hussaini and Abdel-Hamid [6, 7] applied constant-stress ALTs to products whose lifetimes are subject to mixtures of distributions using Bayes and maximum likelihood (ML) estimation methods, respectively. Abdel-Hamid [8] studied the estimation in constant-stress PALTs for Burr XII based on progressive type-II censoring. Ismail [9] derived the ML estimators for the model parameters under constant-stress PALT with Type-II censoring assuming that the lifetime of items at standard stress has Weibull distribution. While in (2012) [10], he studied inference with progressive Type-II censoring for the generalized exponential distribution under PALT. Jaheen et al. [11] applied constant-stress PALTs to products whose lifetimes are subject to the generalized exponential distribution based on progressive Type-II censoring scheme (CS). Ahmad et al. [12] considered constant-stress PALTs when the lifetime of items under use condition follows exponentiated Weibull distribution. Ahmad and Fawzy [13] studied inference in the linear exponential distribution under constant-stress PALTs with progressive Type-II censoring. Shi et al. [14] studied the reliability analysis of hybrid systems with modified Weibull components. Alghamdi [15] applied Type-I generalized hybrid CS for units that fail under two independent causes of failure and units that have Gompertz lifetime distribution. Alam et al. [16] proposed constant-stress PALTs and estimated costs of maintenance service policy for the generalized inverted expo-nential distribution. For more details and some recent references on constant-stress PALT, see [17–20].

The inverted Kumaraswamy (IKum) distribution with two shape parameters *γ* > 0 and *θ* > 0, will be denoted by IKum (*γ*, *θ*). It was derived from Kumaraswamy (Kum) distribution using the transformation *X* = 1/*Y* − 1, when *Y* has a Kum distribution. Three special cases of IKum (*γ*, *θ*) distribution are Lomax distribution (when *θ* = 1), inverted beta Type-II distribution (when *γ* = 1) and log-logistic distribution (in economics it is known as the Fisk distribution) (when *γ* = *θ* = 1). The cumulative distribution function (CDF), probability density function (PDF), and hazard rate function (HRF) of IKum (*γ*, *θ*) are given, respectively, by

$$\begin{array}{ccc} F(x) & = & {\left[1-{\left(1+x\right)}^{-\gamma }\right]}^{\theta },\;x>0\;\text{,}\;\text{(}\gamma ,\theta >0),\\ f(x) & = & \gamma \theta {\left(1+x\right)}^{-(\gamma +1)}{\left[1-{\left(1+x\right)}^{-\gamma }\right]}^{\theta -1},\\ h(x) & = & \frac{\gamma \theta {\left(1+x\right)}^{-(\gamma +1)}{\left[1-{\left(1+x\right)}^{-\gamma }\right]}^{\theta -1}}{1-{\left[1-{\left(1+x\right)}^{-\gamma }\right]}^{\theta }}. \end{array}$$

**Fig 1:**
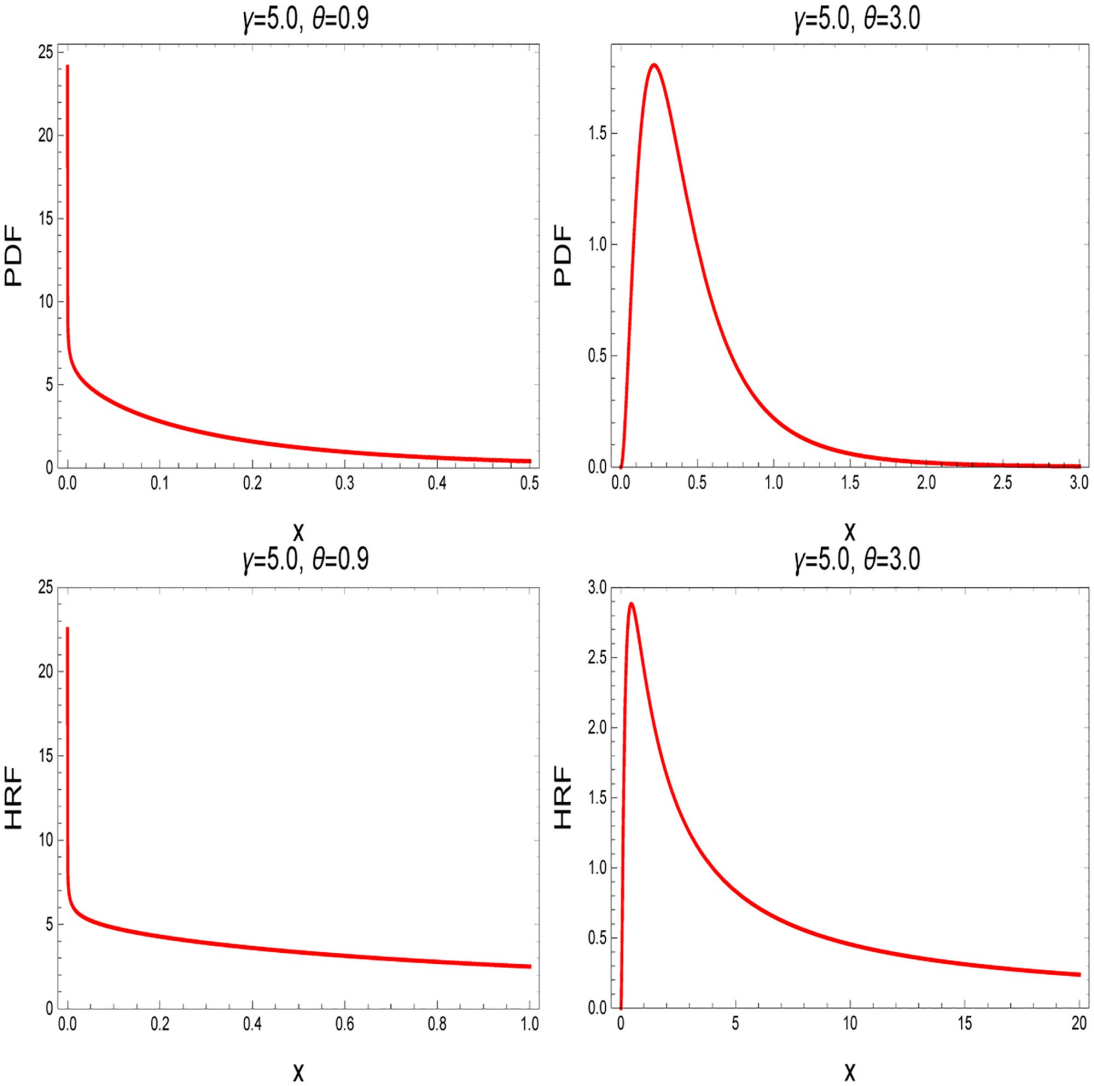
The PDF and HRF of IKum distribution for different values of *γ* and *θ*.

The PDF and HRF curves of IKum distribution show that it has a long right tail when compared to other commonly used distributions, see Fig 1. As a result, it will have an impact on long-term reliability predictions, producing optimistic predictions of rare events that occur in the right tail of the distribution when compared to other distributions. In addition, the IKum distribution fits several data sets in the literature well.

The IKum distribution was proposed by Abd AL-Fattah et al. [21]. They investigated various structural properties with applications. They also addressed the estimation problem of parameters of the IKum distribution based on Type-II censoring. AL-Dayian et al. [22] estimated the shape parameters, reliability function (RF) and HRF of IKum based on dual generalized order statistics. Bagci et al. [23] employed ML, least squares, and maximum product of spacing methodologies in estimating the parameters of the IKum distribution.

Types- I and II CSs are frequently used by the experimenters. A hybrid CS (HCS) is considered a mixture of them. It was introduced by Epstin [24]. One of the drawbacks of the conventional Type-I, Type-II, or HCSs is that they do not permit removal of items at points other than the experiment terminal point. One CS known as the progressive Type-II CS, which permits the experimenter to remove items at several points other than the experiment terminal point, has become very popular in the last few years. Kundu and Joarder [25] proposed a new CS named Type-I progressive HCS (Type-I PHCS) and studied the ML estimates (MLEs) and Bayes estimates for an exponential distribution. The progressive HCS can provide more information about the tail of the distribution under consideration. Furthermore, it can overcome the drawbacks associated with Type-I and Type-II CSs. For example, in Type-II censoring, the experiment termination time is uncontrolled, whereas the efficiency level is controlled in Type-I censoring. For more details and some recent references on CSs, see [26–36].

**Fig 2:**
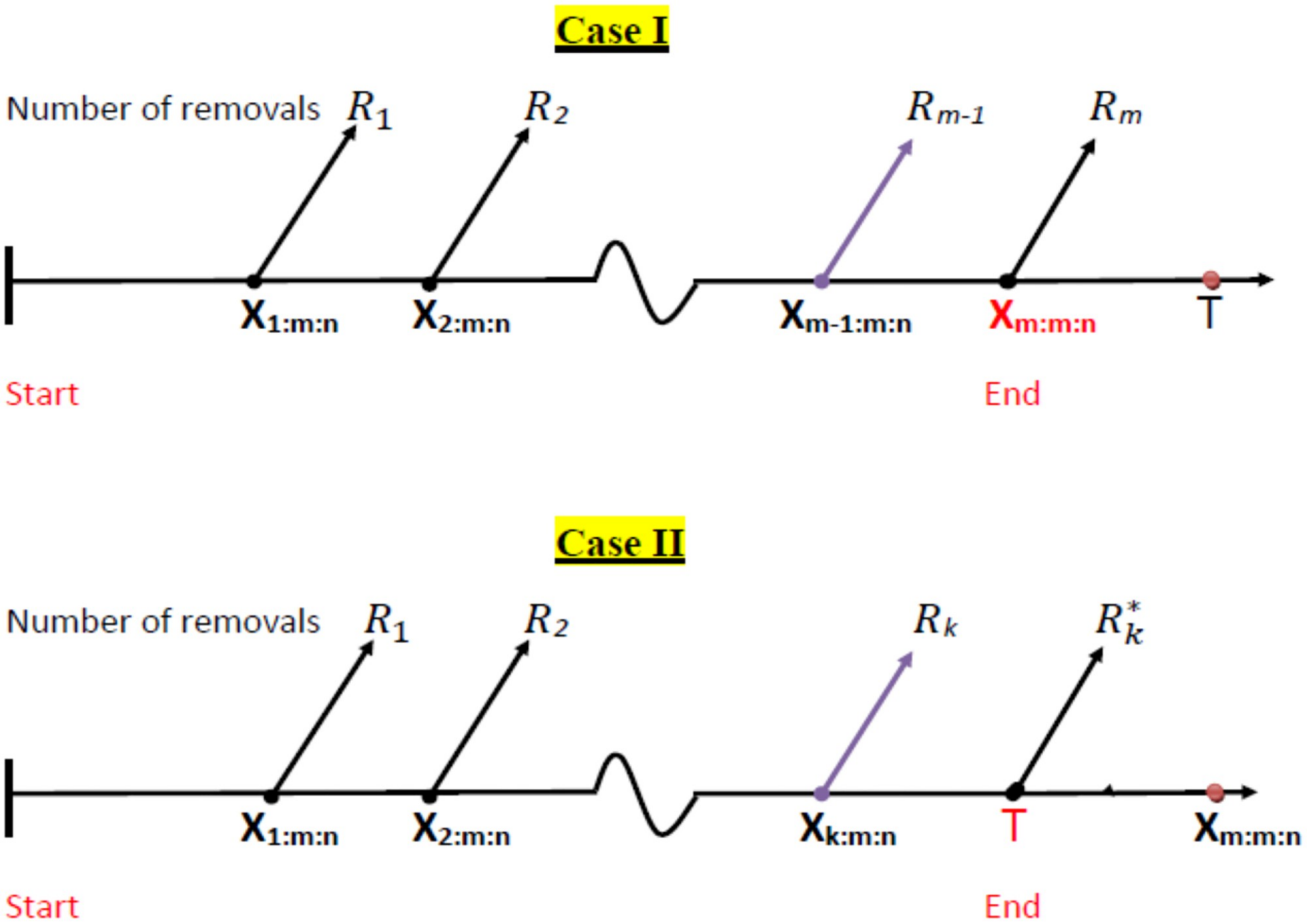
The process of generating order statistics under Type-I PHCS.

Suppose that *n* identical items are placed on a life test. The procedure of progressive Type-II censoring assumes that the integers censoring values *R*_1_, *R*_2_, …, *R*_*m*_, 1 ≤ *m* ≤ *n*, are assigned in advance with *R*_*j*_ ≥ 0 and $\sum_{j=1}^{m}R_{j}+m=n$. At the time of first failure *X*_1:*m*:*n*_, *R*_1_ of the remaining items are randomly removed. Similarly, at the time of the second failure *X*_2:*m*:*n*_, *R*_2_ of the remaining items are removed and so on. Finally at the time of the *m*-th failure *X*_*m*:*m*:*n*_, all remaining *R*_*m*_ = *n* − *R*_1_ − *R*_2_ − … − *R*_*m*−1_ − *m* surviving items are removed from the life test. Hence, *X*_1:*m*:*n*_ < … < *X*_*m*:*m*:*n*_ denote the progressively censored failure times, where *X*_*i*:*m*:*n*_ stands for the *i*-th failure time of the *m* observed failures among *n* tested items, *i* = 1, …, *m*. Let *T* be a prescribed time point. The Type-I PHCS involves the termination of the life test at the time T* = min(*X*_*m*:*m*:*n*_, *T*). If the *m*-th failure occurs before *T*, then the experiment is terminated at the time point *X*_*m*:*m*:*n*_. Otherwise, the experiment stops at time *T* satisfying *X*_*k*:*m*:*n*_ ≤ *T* < *X*_*k*+ 1:*m*:*n*_, and all the remaining $R_{k}^{*}=n-R_{1}-\ldots -R_{k}-k$ live test items are removed, see Fig 2. Here, *k* represents the number of failures occurring up to the time point *T*. We denote these two cases as Case I and Case II, respectively.

Case I: *X*_1:*m*:*n*_ < *X*_2:*m*:*n*_ < … < *X*_*m*:*m*:*n*_, if *X*_*m*:*m*:*n*_ ≤ *T*.

Case II: *X*_1:*m*:*n*_ < *X*_2:*m*:*n*_ < … < *X*_*k*:*m*:*n*_, if *T* < *X*_*m*:*m*:*n*_, *X*_*k*:*m*:*n*_ ≤ *T* < *X*_*k* + 1:*m*:*n*_.

The novelty of this article is to apply the constant-stress PALT to items whose lifetimes under normal stress conditions follow the IKum distribution under Type-I PHCS and to estimate the involved parameters using ML and Bayes (under linear exponential (LINEX), general entropy (GE), and squared error (SE) loss functions) methods. A real data set is analyzed to demonstrate and assess the performance of the introduced estimation methods.

The remaining sections of the article are arranged as follows: In Section 2, the model description is provided. MLEs and asymptotic confidence intervals (CIs) of the unknown parameters are discussed in Section 3. In Section 4, using the Markov chain Monte Carlo (MCMC) method, Bayes estimates, and credible intervals for the parameters are obtained. A simulation study followed by results is presented in Section 5. In Section 6, a real data set is analyzed to demonstrate and assess the performance of the introduced estimation methods given in Sections 3 and 4. Finally, Section 7 is devoted to some concluding remarks.

## 2 Model description

In constant-stress PALT, the total test items are divided into two groups. Let *n*_1_ be the number of items of group 1 that fail at standard stress *s*_0_ with total lifetime *Y* = *T*. The CDF and PDF of an item’s total lifetime *Y* in group 1 are then given, respectively, by

$$\begin{array}{c} \begin{array}{cc} F_{1}\left(y\right) & ={\left[1-{\left(1+y\right)}^{-\gamma }\right]}^{\theta },\;\;y>0\;\text{,}\;\text{(}\gamma ,\theta >0),\\ f_{1}\left(y\right) & =\gamma \theta {\left(1+y\right)}^{-(\gamma +1)}{\left[1-{\left(1+y\right)}^{-\gamma }\right]}^{\theta -1}. \end{array}\} \end{array}$$ (1)

The life testing experiment is finished at time *T*_1_ when *T*_1_ is reached before occurring the *m*_1_-th failure. Otherwise, the experiment is finished as soon as the *m*_1_-th failure occurs, i.e., the end time is a random variable

$$T_{1}^{*}=\text{min}\left\{Y_{1\;m_{1}:m_{1}:n_{1}},T_{1}\right\}.$$

Let *n*_2_ be the number of items of group 2 that fail at accelerated stress *s*_1_ with total lifetime *Y* = λ^−1^*T*, where *T* is the lifetime of an item at standard stress and λ is an acceleration factor (λ > 1). The CDF and PDF of an item’s total lifetime *Y* in group 2 are then given, respectively, by

$$\begin{array}{c} \begin{array}{cc} F_{2}\left(y\right) & ={\left[1-{\left(1+\lambda y\right)}^{-\gamma }\right]}^{\theta },y>0,\left(\gamma ,\theta >0,\lambda >1\right),\\ f_{2}\left(y\right) & =\gamma \theta \lambda {\left(1+\lambda y\right)}^{-(\gamma +1)}{\left[1-{\left(1+\lambda y\right)}^{-\gamma }\right]}^{\theta -1}. \end{array}\} \end{array}$$ (2)

The life testing experiment is finished at time *T*_2_ when *T*_2_ is achieved before occurring the *m*_2_-th failure. Otherwise, the experiment is finished as soon as the *m*_2_-th failure occurs, i.e., the end time is a random variable

$$T_{2}^{*}=\text{min}\left\{Y_{2\;m_{2}:m_{2}:n_{2}},T_{2}\right\}.$$

Therefore, under Type-I PHCS, for *j* = 1, 2, we have the next two cases:

Case I: $\;Y_{j\;1:m_{j}:n_{j}}\;<\;\ldots \;<\;Y_{j\;m_{j}:m_{j}:n_{j}}$ if $\;Y_{j\;m_{j}:m_{j}:n_{j}}\leq T_{j}.$

Case II $:\;\;\;\;Y_{j\;1:m_{j}:n_{j}}<\ldots <Y_{j\;k_{j}:m_{j}:n_{j}}$ if $T_{j}<Y_{j\;m_{j}:m_{j}:n_{j}},$ $\;Y_{j\;k_{j}:m_{j}:n_{j}}\leq T_{j}<Y_{j\;k_{j+1}:m_{j}:n_{j}}.$

## 3 Maximum likelihood estimation

In this section, we construct the likelihood function (LF), based on observed data that are subject to Type-I PHCS, to obtain ML estimators of the parameters *γ*, *θ*, and λ. From CDF (1) and its corresponding PDF (2), we obtain the LFs of *γ*, *θ* and λ.

The LF for group 1 is as follows, see [25],

$$\begin{array}{cc} L(\gamma ,\theta |y_{1}) & =C_{1}\prod_{i=1}^{D_{1}}f_{1}\left(y_{1i:m_{1}:n_{1}}\right){\left[1-F_{1}\left(y_{1i:m_{1}:n_{1}}\right)\right]}^{R_{1i}}{\left[1-F_{1}\left(T_{1}\right)\right]}^{R_{D_{1}}^{*}}\\ & =C_{1}\prod_{i=1}^{D_{1}}\gamma \theta {\left(1+y_{1i:m_{1}:n_{1}}\right)}^{-(\gamma +1)}{\left[1-{\left(1+y_{1i:m_{1}:n_{1}}\right)}^{-\gamma }\right]}^{\theta -1}\\ & \times {\left\{1-{\left[1-{\left(1+y_{1i:m_{1}:n_{1}}\right)}^{-\gamma }\right]}^{\theta }\right\}}^{R_{1i}}{\left\{1-{\left[1-{\left(1+T_{1}\right)}^{-\gamma }\right]}^{\theta }\right\}}^{R_{D_{1}}^{*}}, \end{array}$$ (3)

where

$$\begin{array}{c} D_{1}=\{\begin{array}{c} m_{1}\text{,}\;\text{for}\;\text{Case}\;\text{I}\;\\ k_{1}\text{,}\;\text{for}\;\text{Case}\;\text{II} \end{array},R_{D_{1}}^{*}=n_{1}-D_{1}-\sum_{j=1}^{D_{1}}R_{j}, \end{array}$$ (4)

*C*_1_ = *n*_1_(*n*_1_ − *R*_1_ − 1)…(*n*_1_ − *R*_1_ − *R*_2_ − … − *R*_*m*_1_−1_ − *D*_1_ + 1) and $y_{1}=\left(y_{11},y_{12},\ldots ,y_{1D_{1}}\right)$, $y_{1i}\equiv y_{1i:m_{1}:n_{1}}.$

The LF for group 2 is as follows:

$$\begin{array}{cc} L(\gamma ,\theta ,\lambda |y_{2})= & C_{2}\prod_{i=1}^{D_{2}}\gamma \theta \lambda {\left(1+\lambda y_{2i:m_{2}:n_{2}}\right)}^{-(\gamma +1)}{\left[1-{\left(1+\lambda y_{2i:m_{2}:n_{2}}\right)}^{-\gamma }\right]}^{\theta -1}\\ & \times {\left\{1-{\left[1-{\left(1+\lambda y_{2i:m_{2}:n_{2}}\right)}^{-\gamma }\right]}^{\theta }\right\}}^{R_{2i}}{\left\{1-{\left[1-{\left(1+\lambda T_{2}\right)}^{-\gamma }\right]}^{\theta }\right\}}^{R_{D_{2}}^{*}}, \end{array}$$ (5)

where

$$\begin{array}{c} D_{2}=\{\begin{array}{c} m_{2}\text{,}\;\text{for}\;\text{Case}\;\text{I}\;\\ k_{2}\text{,}\;\text{for}\;\text{Case}\;\text{II} \end{array},R_{D_{2}}^{*}=n_{2}-D_{2}-\sum_{j=1}^{D_{2}}R_{j}, \end{array}$$ (6)

*C*_2_ = *n*_2_(*n*_2_ − *R*_1_ − 1)…(*n*_2_ − *R*_1_ − *R*_2_ − … − *R*_*m*_2_−1_ − *D*_2_ + 1) and $y_{2}=\left(y_{21},y_{22},\ldots ,y_{2D_{2}}\right),$ $y_{2i}\equiv y_{2i:m_{2}:n_{2}}.$

Based on (4) and (6), Eqs (3) and (5) could be written in one equation as follows:

$$\begin{array}{cc} L(\gamma ,\theta ,\lambda |y) & =\prod_{j=1}^{2}C_{j}\prod_{i=1}^{D_{j}}\gamma \theta \lambda^{j-1}{\left(1+\lambda^{j-1}y_{ji:m_{j}:n_{j}}\right)}^{-(\gamma +1)}{\left[1-{\left(1+\lambda^{j-1}y_{ji:m_{j}:n_{j}}\right)}^{-\gamma }\right]}^{\theta -1}\\ & \times {\left\{1-{\left[1-{\left(1+\lambda^{j-1}y_{ji:m_{j}:n_{j}}\right)}^{-\gamma }\right]}^{\theta }\right\}}^{R_{ji}}{\left\{1-{\left[1-{\left(1+\lambda^{j-1}T_{j}\right)}^{-\gamma }\right]}^{\theta }\right\}}^{R_{D_{j}}^{*}}. \end{array}$$ (7)

The logarithm of LF (7) can be written as

$$\begin{array}{cc} l(\gamma ,\theta ,\lambda ) & \propto \sum_{j=1}^{2}[D_{j}\text{ln}\;\gamma \theta +\left(j-1\right)D_{j}\text{ln}\lambda -\left(\gamma +1\right)\sum_{i=1}^{D_{j}}\text{ln}(1+\lambda^{j-1}y_{ji:m_{j}:n_{j}})+\left(\theta -1\right)\\ & \times \sum_{i=1}^{D_{j}}\text{ln}[1-{\left(1+\lambda^{j-1}y_{ji:m_{j}:n_{j}}\right)}^{-\gamma }]+\sum_{i=1}^{D_{j}}R_{ji}\text{ln}[1-{\left\{1-{\left(1+\lambda^{j-1}y_{ji:m_{j}:n_{j}}\right)}^{-\gamma }\right\}}^{\theta }]\\ & +R_{D_{j}}^{*}\text{ln}[1-{\left\{1-{\left(1+\lambda^{j-1}T_{j}\right)}^{-\gamma }\right\}}^{\theta }]]. \end{array}$$ (8)

To evaluate the ML estimators of (*γ*, *θ*, λ), we equate to zero the first partial derivatives of (8) with respect to *γ*, *θ*, and λ as follows:

$$\begin{array}{c} \begin{array}{cc} \frac{\partial l}{\partial \gamma }=0= & \frac{D_{1}+D_{2}}{\gamma }-\sum_{j=1}^{2}[\sum_{i=1}^{D_{j}}(\text{ln}\;G_{j}\left(\lambda^{j-1},y_{ji:m_{j}:n_{j}}\right)+\frac{\left\{1-\theta +\theta R_{ji}G_{2j}\left(\lambda^{j-1},y_{ji:m_{j}:n_{j}}\right)\right\}}{G_{1j}\left(\lambda^{j-1},y_{ji:m_{j}:n_{j}}\right)}\\ & \times \frac{\partial G_{1j}\left(\lambda^{j-1},y_{ji:m_{j}:n_{j}}\right)}{\partial \gamma })+\theta R_{D_{j}}^{*}\frac{G_{2j}\left(\lambda^{j-1},T_{j}\right)}{G_{1j}\left(\lambda^{j-1},T_{j}\right)}\frac{\partial G_{1j}\left(\lambda^{j-1},T_{j}\right)}{\partial \gamma }],\\ \frac{\partial l}{\partial \theta }=0= & \frac{D_{1}+D_{2}}{\theta }+\sum_{j=1}^{2}[\sum_{i=1}^{D_{j}}(\left\{1-R_{ji}G_{2j}\left(\lambda^{j-1},y_{ji:m_{j}:n_{j}}\right)\right\}\text{ln}G_{1j}\left(\lambda^{j-1},y_{ji:m_{j}:n_{j}}\right))\\ & -R_{D_{j}}^{*}G_{2j}\left(\lambda^{j-1},T_{j}\right)\text{ln}G_{1j}\left(\lambda^{j-1},T_{j}\right)],\\ \frac{\partial l}{\partial \lambda }=0= & \frac{D_{2}}{\lambda }-\sum_{i=1}^{D_{2}}[\frac{\left(\gamma +1\right)y_{2i:m_{2}:n_{2}}}{G_{2}\left(\lambda ,y_{2i:m_{2}:n_{2}}\right)}+\frac{\left\{1-\theta +\theta R_{2i}G_{22}\left(\lambda ,y_{2i:m_{2}:n_{2}}\right)\right\}}{G_{12}\left(\lambda ,y_{2i:m_{2}:n_{2}}\right)}\frac{\partial G_{12}\left(\lambda ,y_{2i:m_{2}:n_{2}}\right)}{\partial \lambda }]\\ & -\theta R_{D_{2}}^{*}\frac{G_{22}\left(\lambda ,T_{2}\right)}{G_{12}\left(\lambda ,T_{2}\right)}\frac{\partial G_{12}\left(\lambda ,T_{2}\right)}{\partial \lambda }, \end{array}\} \end{array}$$ (9)

where

$$\begin{array}{c} \begin{array}{cc} G_{j}\left(\lambda^{j-1},y_{ji:m_{j}:n_{j}}\right) & =(1+\lambda^{j-1}y_{ji:m_{j}:n_{j}}),\\ G_{1j}\left(\lambda^{j-1},y_{ji:m_{j}:n_{j}}\right) & =1-{\left[G_{j}\left(\lambda^{j-1},y_{ji:m_{j}:n_{j}}\right)\right]}^{-\gamma },\\ G_{2j}\left(\lambda^{j-1},y_{ji:m_{j}:n_{j}}\right) & =\frac{{\left[G_{1j}\left(\lambda^{j-1},y_{ji:m_{j}:n_{j}}\right)\right]}^{\theta }}{1-{\left[G_{1j}\left(\lambda^{j-1},y_{ji:m_{j}:n_{j}}\right)\right]}^{\theta }},\\ \frac{\partial G_{1j}\left(\lambda^{j-1},y_{ji:m_{j}:n_{j}}\right)}{\partial \gamma } & ={\left[G_{j}\left(\lambda^{j-1},y_{ji:m_{j}:n_{j}}\right)\right]}^{-\gamma }\text{ln}\;G_{j}\left(\lambda^{j-1},y_{ji:m_{j}:n_{j}}\right),\\ \frac{\partial G_{12}\left(\lambda ,y_{2i:m_{2}:n_{2}}\right)}{\partial \lambda } & =\gamma y_{2i:m_{2}:n_{2}}{\left[G_{2}\left(\lambda ,y_{2i:m_{2}:n_{2}}\right)\right]}^{-(\gamma +1)}, \end{array}\} \end{array}$$ (10)

and *G*_1*j*_(λ^*j*−1^, *T*_*j*_) is as given in (10) with $T_{j}=y_{ji:m_{j}:n_{j}}$. By the same way for *G*_2*j*_(λ^*j*−1^, *T*_*j*_).

The ML estimators can be got by solving the nonlinear equations given in (9) with respect to (*γ*, *θ*, λ). It is clear that system of nonlinear equations (9) is not in an analytically tractable form. Therefore, a suitable numerical method must be applied to get MLEs of *γ*, *θ*, and λ. An iterative algorithm such as the Newton-Raphson can be utilized to get the MLEs $\hat{\gamma }$, $\hat{\theta }$ and $\hat{\lambda }$.

### 3.1 Asymptotic confidence interval

In this subsection, the approximate CIs of the unknown parameters (*γ*, *θ*, λ) are deduced based on the asymptotic distribution of the MLEs.

The observed Fisher information matrix, **I**, is a symmetric matrix of the second negative partial derivatives of the logarithm of LF. Therefore, **I** is given by, see Nelson [1],

$$I\left(\hat{\gamma },\hat{\theta },\hat{\lambda }\right)=-(\frac{\partial^{2}l\left(\gamma ,\theta ,\lambda \right)}{\partial \alpha_{p}\partial \alpha_{q}}){|}_{{}_{\left(\hat{\gamma },\hat{\theta },\hat{\lambda }\right)}},\;p,q=1,2,3,\;\alpha_{1}=\gamma ,\alpha_{2}=\theta ,\alpha_{3}=\lambda ,$$

where the second partial derivatives included in **I** are given in S1 Appendix.

The asymptotic variance-covariance matrix may be approximated as the inverse of **I**. That is,

$$I^{-1}\left(\hat{\gamma },\hat{\theta },\hat{\lambda }\right)={\left(\text{cov}\left({\hat{\alpha }}_{p},{\hat{\alpha }}_{q}\right)\right)}_{3\times 3},p,q=1,2,3.$$

The asymptotic distribution of the MLE of (*γ*, *θ*, λ) is given by, see Miller [37],

$$[\left(\gamma -\hat{\gamma }\right),\left(\theta -\hat{\theta }\right),\left(\lambda -\hat{\lambda }\right)]∼N_{3}(0,I^{-1}\left(\hat{\gamma },\hat{\theta },\hat{\lambda }\right)).$$

Based on the normal approximation CI (NACI), the two-sided 100(1 − *ϵ*)% CIs for *γ*, *θ* and λ are given by

$$(\hat{\gamma }\mp \;z_{1-\epsilon /2}\sqrt{\text{var}(\hat{\gamma })}),(\hat{\theta }\mp \;z_{1-\epsilon /2}\sqrt{\text{var}(\hat{\theta })})\;\text{and}\;(\hat{\lambda }\mp \;z_{1-\epsilon /2}\sqrt{\text{var}(\hat{\lambda })}),$$

where $z_{1-\in /2}$ is the upper (*ϵ*/2) percentile of the standard normal distribution.

The NACIs obtained above may occasionally have negative lower bounds. To overcome this problem, Meeker and Escobar [38], and Xu and Long [39] considered approximate CI for the parameters using the log-transformation and delta approaches. Two-sided 100(1 − *ϵ*)% normal approximation, based on log-transformed ML estimators, CIs (LTCIs) for *γ*, *θ* and λ are then given by

$$\begin{array}{ccc} & & (\hat{\gamma }\;\text{exp}[-(z_{1-\epsilon /2}\sqrt{\text{var}(\hat{\gamma })})/\hat{\gamma }]\text{,}\;\;\hat{\gamma }\;\text{exp}[(z_{1-\epsilon /2}\sqrt{\text{var}(\hat{\gamma })})/\hat{\gamma }]),\\ & & (\hat{\theta }\;\text{exp}[-(z_{1-\epsilon /2}\sqrt{\text{var}(\hat{\theta })})/\hat{\theta }]\text{,}\;\;\hat{\theta }\;\text{exp}[(z_{1-\epsilon /2}\sqrt{\text{var}(\hat{\theta })})/\hat{\theta }]),\\ & & (\hat{\lambda }\;\text{exp}[-(z_{1-\epsilon /2}\sqrt{\text{var}(\hat{\lambda })})/\hat{\lambda }]\text{,}\;\;\hat{\lambda }\;\text{exp}[(z_{1-\epsilon /2}\sqrt{\text{var}(\hat{\lambda })})/\hat{\lambda }]). \end{array}$$

## 4 Bayes estimation

In Bayes analysis, MCMC method is a general computing method applicable to both non-informative and informative priors. It makes Bayesian analysis attractive for a complex model. In the current section, we discuss the Bayes estimators of the parameters under the assumptions of informative and non-informative priors for *γ*, *θ* and λ. Bayes estimators are considered under LINEX, GE, and SE loss functions as we present below.

Since the invariance property does not hold with the Bayes estimation method, then the Bayes estimators of the parameters *γ*, *θ* and λ, in addition to the RF and HRF should be calculated separately.

For simplicity, assume that *G* = *G*(*α*) ≡ *G*(*γ*, *θ*, λ) is a function of vector of parameters to be estimated. Clearly, *G* may assume one of the parameters or a combination of them (such as *G*≡ RF or *G*≡ HRF). Then the BE of *G* based on LINEX, GE, and SE loss functions are given by, see [40–42],

$$\begin{array}{c} {\hat{G}}_{BL}=-\frac{1}{c}\text{ln}(\text{E}[e^{-c\;G(\alpha )}|y])=-\frac{1}{c}\text{ln}(\int_{\alpha }e^{-cG(\alpha )}\pi^{*}\left(\alpha |y\right)\text{d}\alpha ),c\neq 0,\\ {\hat{G}}_{BG}={\left(\text{E}[{\left\{G\left(\alpha \right)\right\}}^{-c}|y]\right)}^{-1/c}={\left(\int_{\alpha }{\left\{G\left(\alpha \right)\right\}}^{-c}\pi^{*}\left(\alpha |y\right)\text{d}\alpha \right)}^{-1/c},c\neq 0,\\ {\hat{G}}_{BS}=\text{E}\left[G\left(\alpha \right)|y\right]=\int_{\alpha }G\left(\alpha \right)\pi^{*}\left(\alpha |y\right)\text{d}\alpha . \end{array}$$

Assume that the experimenter’s prior belief is described by a function *π*(*γ*, *θ*, λ) and that the parameters *γ*, *θ* and λ are independent random variables. Let *π*_1_(*γ*) and *π*_2_(*θ*), respectively, denote the prior densities for *γ* and *θ*, following gamma distributions and let *π*_3_(λ) denote a non-informative prior for λ, with respective densities given by,

$$\begin{array}{c} \pi_{1}\left(\gamma \right)\propto \gamma^{\mu -1}e^{-\gamma /\nu },\left(\gamma ,\mu ,\nu \right)>0,\\ \pi_{2}\left(\theta \right)\propto \theta^{\zeta -1}e^{-\theta /\eta },\left(\theta ,\zeta ,\eta \right)>0,\\ \pi_{3}\left(\lambda \right)\propto \lambda^{-1},\lambda >1. \end{array}$$

Therefore, the joint prior distribution of *γ*, *θ* and λ can be written as

$$\begin{array}{c} \pi \left(\gamma ,\theta ,\lambda \right)\propto \gamma^{\mu -1}\theta^{\zeta -1}\lambda^{-1}e^{-(\gamma /\nu +\theta /\eta )}. \end{array}$$ (11)

The informative gamma prior distribution is used in this article because it is more appropriate than non-informative prior. Merging LF (7) with joint prior distribution of *γ*, *θ* and λ (11), we get the joint posterior density of *γ*, *θ* and λ, given the data, as follows:

$$\begin{array}{cc} \pi^{*}\left(\gamma ,\theta ,\lambda ;y\right) & \propto \gamma^{D_{1}+D_{2}+\mu -1}\theta^{D_{1}+D_{2}+\zeta -1}\lambda^{D_{2}-1}e^{-(\gamma /\nu +\theta /\eta )}\prod_{j=1}^{2}\prod_{i=1}^{D_{j}}{\left(1+\lambda^{j-1}y_{ji:m_{j}:n_{j}}\right)}^{-(\gamma +1)}\\ & \times {\left[1-{\left(1+\lambda^{j-1}y_{ji:m_{j}:n_{j}}\right)}^{-\gamma }\right]}^{\theta -1}{\left\{1-{\left[1-{\left(1+\lambda^{j-1}y_{ji:m_{j}:n_{j}}\right)}^{-\gamma }\right]}^{\theta }\right\}}^{R_{ji}}\\ & \times {\left\{1-{\left[1-{\left(1+\lambda^{j-1}T_{j}\right)}^{-\gamma }\right]}^{\theta }\right\}}^{R_{D_{j}}^{*}}. \end{array}$$ (12)

From (12), it is obvious that the Bayes estimators of *γ*, *θ* and λ cannot be got in closed forms. Therefore, we adopt MCMC method to obtain them.

### 4.1 Markov chain Monte Carlo

MCMC has an important role in Bayesian inference. The Gibbs sampling and Metropolis-Hastings algorithms are the two most widely used MCMC methods that are applied in statistics, statistical physics, signal processing, digital communications, machine learning, etc.

Using joint posterior density function (12), the conditional posterior distributions of model parameters *γ*, *θ*, λ can be written, respectively, in the forms

$$\begin{array}{c} \begin{array}{cc} \pi^{*}\left(\gamma |\theta ,\lambda ,y\right) & \propto \gamma^{D_{1}+D{}_{2}+\mu -1}e^{-\gamma /\nu }\prod_{j=1}^{2}\prod_{i=1}^{D_{j}}{\left(1+\lambda^{j-1}y_{ji:m_{j}:n_{j}}\right)}^{-\gamma }{\left[1-{\left(1+\lambda^{j-1}y_{ji:m_{j}:n_{j}}\right)}^{-\gamma }\right]}^{\theta -1},\\ & \times {\left\{1-{\left[1-{\left(1+\lambda^{j-1}y_{ji:m_{j}:n_{j}}\right)}^{-\gamma }\right]}^{\theta }\right\}}^{R_{ji}}{\left\{1-{\left[1-{\left(1+\lambda^{j-1}T_{j}\right)}^{-\gamma }\right]}^{\theta }\right\}}^{R_{D_{j}}^{*}},\\ \pi^{*}\left(\theta |\gamma ,\lambda ,y\right) & \propto \theta^{D_{1}+D{}_{2}+\zeta -1}e^{-\theta /\eta }\prod_{j=1}^{2}\prod_{i=1}^{D_{j}}{\left[1-{\left(1+\lambda^{j-1}y_{ji:m_{j}:n_{j}}\right)}^{-\gamma }\right]}^{\theta }\\ & \times {\left\{1-{\left[1-{\left(1+\lambda^{j-1}y_{ji:m_{j}:n_{j}}\right)}^{-\gamma }\right]}^{\theta }\right\}}^{R_{ji}}{\left\{1-{\left[1-{\left(1+\lambda^{j-1}T_{j}\right)}^{-\gamma }\right]}^{\theta }\right\}}^{R_{D_{j}}^{*}},\\ \pi^{*}\left(\lambda |\gamma ,\theta ,y\right) & \propto \lambda^{D{}_{2}-1}\prod_{i=1}^{D_{2}}{\left(1+\lambda y_{2i:m_{2}:n_{2}}\right)}^{-(\gamma +1)}{\left[1-{\left(1+\lambda y_{2i:m_{2}:n_{2}}\right)}^{-\gamma }\right]}^{\theta -1}\\ & \times {\left\{1-{\left[1-{\left(1+\lambda y_{2i:m_{2}:n_{2}}\right)}^{-\gamma }\right]}^{\theta }\right\}}^{R_{2i}}{\left\{1-{\left[1-{\left(1+\lambda T_{2}\right)}^{-\gamma }\right]}^{\theta }\right\}}^{R_{D_{2}}^{*}}. \end{array} \end{array}$$ (13)

The conditional posterior PDFs of *γ*, *θ*, and λ do not belong to well-known distributions, and hence generating samples from (13) is not available, directly. Consequently, with normal proposal distribution, Metropolis-Hastings algorithm is applied to get Bayesian estimates for *γ*, *θ*, and λ.

The following MCMC procedure is suggested to evaluate the Bayes estimates of *γ*, *θ*, and λ and the corresponding credible intervals.

- Step 1. Select the MLEs $\hat{\gamma },\hat{\theta }$ and $\hat{\lambda }$, as starting values (*γ*_(0)_, *θ*_(0)_, λ_(0)_) for *γ*, *θ* and λ.
- Step 2. Set *i* = 1.
- Step 3. Generate *γ*_(*i*)_ from π*(*γ*|*θ*_(*i* − 1)_, λ_(*i* − 1)_, **y**).
- Step 4. Generate *θ*_(*i*)_ from π*(*θ*|*γ*_(*i* − 1)_, λ_(*i* − 1)_, **y**).
- Step 5. Generate λ_(*i*)_ from π*(λ|*γ*_(*i* − 1)_, *θ*_(*i* − 1)_, **y**).
- Step 6. Set *i* = *i* + 1.
- Step 7. Iterate Steps 3–6 *N* times.
- Step 8. Get the Bayes estimates of *γ*, *θ*, and λ based on SE loss function as
  $$\begin{array}{c} \begin{array}{c} {\hat{\gamma }}_{BS}=\frac{1}{N-M}\sum_{i=M+1}^{N}\gamma_{\left(i\right)},{\hat{\theta }}_{BS}=\frac{1}{N-M}\sum_{i=M+1}^{N}\theta_{\left(i\right)},{\hat{\lambda }}_{BS}=\frac{1}{N-M}\sum_{i=M+1}^{N}\lambda_{\left(i\right)}, \end{array} \end{array}$$
  where *M* is the burn-in period.
- Step 9. Get the Bayes estimates of *γ*, *θ*, and λ based on LINEX loss function as
  $$\begin{array}{c} \begin{array}{cc} {\hat{\gamma }}_{BL} & =\frac{-1}{c}\text{ln}(\frac{1}{N-M}\sum_{i=M+1}^{N}e^{-c\gamma_{\left(i\right)}}),\\ {\hat{\theta }}_{BL} & =\frac{-1}{c}\text{ln}(\frac{1}{N-M}\sum_{i=M+1}^{N}e^{-c\theta_{\left(i\right)}}),\\ {\hat{\lambda }}_{BL} & =\frac{-1}{c}\text{ln}(\frac{1}{N-M}\sum_{i=M+1}^{N}e^{-c\lambda_{\left(i\right)}}). \end{array} \end{array}$$
- Step 10. Get the Bayes estimates of *γ*, *θ*, and λ based on GE loss function as
  $$\begin{array}{c} \begin{array}{cc} {\hat{\gamma }}_{BG} & ={\left(\frac{1}{N-M}\sum_{i=M+1}^{N}{\left[\gamma_{\left(i\right)}\right]}^{-c}\right)}^{-1/c},\\ {\hat{\theta }}_{BG} & ={\left(\frac{1}{N-M}\sum_{i=M+1}^{N}{\left[\theta_{\left(i\right)}\right]}^{-c}\right)}^{-1/c},\\ {\hat{\lambda }}_{BG} & ={\left(\frac{1}{N-M}\sum_{i=M+1}^{N}{\left[\lambda_{\left(i\right)}\right]}^{-c}\right)}^{-1/c}. \end{array} \end{array}$$
- Step 11: To calculate the credible intervals for *γ*, order *γ*_(*M*+ 1)_, …, *γ*_(*N*)_ as *γ*_[1]_ < … < *γ*_[*N*−*M*]_. Then the 100(1 − *ϵ*)% credible interval for *γ* is given by
  $$\begin{array}{c} (\gamma_{\left[(N-M)\epsilon /2\right]},\gamma_{\left[(N-M)(1-\epsilon /2)\right]}). \end{array}$$
  [*w*] denotes the greatest integer less than or equal to *w*
  Similarly, it is also possible to construct credible intervals for *θ* and λ.

## 5 Simulation study

We are performing a simulation analysis in this section to compare the performance of various estimates and CIs. The different estimates (MLE and Bayes estimates) are compared in terms of mean squared errors (MSEs) and relative absolute biases (RABs). Also, the different intervals (asymptotic and credible intervals) are compared in terms of average lengths and coverage probabilities. The simulation study is done according to the following procedure.

**Steps to proceed the simulation study:**

- Step 1: Assign values for *n*_1_, *n*_2_, *m*_1_, *m*_2_, *T*_1_, *T*_2_, λ, *μ*, *ν*, *ζ*, *η*, *c* and CSs *R*_*ji*_, *i* = 1, …, *m*_*j*_, *j* = 1, 2.
- Step 2: For given values of the prior parameters *μ*, *ν*, *ζ* and *η*, generate *γ* and *θ* from gamma (*μ*, *ν*) and gamma(*ζ*, *η*) distributions.
- Step 3: Generate two random samples from CDFs *F*_1_(*y*) and *F*_2_(*y*), given in (1) and (2), respectively, and then apply the progressive hybrid censoring technique to them to generate the two samples $y_{j1},\ldots ,y_{jD_{j}}$, *j* = 1, 2.
- Step 4: The MLEs of the parameters *γ*, *θ*, λ are got by solving nonlinear equations (8) using the Newton-Raphson technique.
- Step 5: The approximate CIs are established, via the matrix of asymptotic variance-covariance of the estimators.
- Step 6: Apply the Metropolis-Hastings algorithm to generate an iterative sequence of 11000 of random samples with *N* = 11000 and *M* = 1000.
- Step 7: The Bayes estimates of (*γ*, *θ*, λ) are calculated based on the LINEX, GE, and SE loss functions.
- Step 8: The squared deviations (γ* − *γ*)^2^, (θ* − *θ*)^2^ and (λ* − λ)^2^ are calculated where γ*, θ*, and λ* are estimates of *γ*, *θ*, and λ.
- Step 9: Steps 2–8 are iterated 1000 times for various sample sizes and various CSs. MSEs and RABs of all the estimates are calculated.

Table 1 presents the various CSs, applied in the simulation study, for various choices of sample sizes *n*_*j*_, *j* = 1, 2, and observed failure times *m*_*j*_ which describe 60% and 80% of the sample size. In Tables 2, 3, 5 and 6, we provide the MSEs and RABs of ML and Bayes estimates for the parameters *γ*, *θ* and λ relative to SE, LINEX and GE (with different values of *c*) loss functions, considering *T*_1_ = 0.9, and *T*_2_ = 0.5. The average interval lengths (AILs) and the corresponding 95% coverage probabilities (CPs), using the asymptotic distributions of MLEs, and credible intervals are displayed in Tables 4 and 7. The prior parameters have been chosen to be *μ* = 2, *v* = 1.5, ζ = 1.5 *η* = 1.5, which yield the generated values γ = 1.85699 and *θ* = 2.50899 (as true values) with λ = 1.5, considering some different Type-I PHCSs as in Table 1 with notation that (3 * 0, 1) means (0, 0, 0, 1).

**Table 1:** Progressive CSs that have been used in the Monte Carlo simulation.

(*n*_1_, *m*_1_) (*n*_2_, *m*_2_)	CS	$\left(R_{1},R_{2},...,R_{m_{1}}\right)$ $\left(R_{1},R_{2},...,R_{m_{2}}\right)$
(30,18) (40,24)	I	*R*_1*i*_ = (12, 17 * 0)
		*R*_2*i*_ = (16, 23 * 0)
	II	*R*_1*i*_ = (6, 16 * 0, 6)
		*R*_2*i*_ = (8, 22 * 0, 8)
	III	*R*_1*i*_ = (2, 0, 2, 0, 2, 0, 2, 0, 4, 9 * 0)
		*R*_2*i*_ = (2, 0, 2, 0, 2, 0, 2, 0, 2, 0, 2, 0, 4, 11 * 0)
(30,24) (40,32)	I	*R*_1*i*_ = (6, 23 * 0)
		*R*_2*i*_ = (8, 31 * 0)
	II	*R*_1*i*_ = (3, 22 * 0, 3)
		*R*_2*i*_ = (4, 30 * 0, 4)
	III	*R*_1*i*_ = (1, 0, 1, 0, 1, 0, 3, 17 * 0)
		*R*_2*i*_ = (1, 0, 1, 0, 1, 0, 1, 0, 1, 0, 3, 21 * 0)
(60,36) (70,42)	I	*R*_1*i*_ = (24, 35 * 0)
		*R*_2*i*_ = (28, 41 * 0)
	II	*R*_1*i*_ = (12, 34 * 0, 12)
		*R*_2*i*_ = (14, 40 * 0, 14)
	III	*R*_1*i*_ = (2, 0, 2, 0, 2, 0, 2, 0, 2, 0, 2, 0, 2, 0, 2, 0, 2, 0, 2, 0, 4, 0, 15 * 0)
		*R*_2*i*_ = (2, 0, 2, 0, 2, 0, 2, 0, 2, 0, 2, 0, 2, 0, 2, 0, 2, 0, 2, 0, 2, 0, 2, 0, 4, 0, 17 * 0)
(60,48) (70,56)	I	*R*_1*i*_ = (12, 47 * 0)
		*R*_2*i*_ = (14, 55 * 0)
	II	*R*_1*i*_ = (6, 46 * 0, 6)
		*R*_2*i*_ = (7, 54 * 0, 7)
	III	*R*_1*i*_ = (1, 0, 1, 0, 1, 0, 1, 0, 1, 0, 1, 0, 1, 0, 1, 0, 1, 0, 3, 29 * 0)
		*R*_2*i*_ = (1, 0, 1, 0, 1, 0, 1, 0, 1, 0, 1, 0, 1, 0, 1, 0, 1, 0, 1, 0, 1, 0, 3, 33 * 0)

**Table 2:** MSEs for the MLEs and Bayes estimates of (*γ*, *θ*, λ) for different schemes with *T*_1_ = 0.9, *T*_2_ = 0.5. Prior parameter values are: *μ* = 2, *ν* = 1.5, *ζ* = 1.5, *η* = 1.5. Population parameter values are: *γ* = 1.85699, *θ* = 2.50899, λ = 1.5.

(*n*_1_, *m*_2_) (*n*_2_, *m*_2_)	CS	$\hat{\alpha }$	ML	Bayes						
				SE	LINEX			GE		
					*c* = −0.5	*c* = 0.5	*c* = 2.0	*c* = −0.5	*c* = 0.5	*c* = 2.0
(30,18) (40,24)	I	*γ*	0.37651	0.22012	0.25051	0.20214	0.20281	0.21755	0.22184	0.25686
		*θ*	1.1923	0.53333	0.85754	0.38544	0.29718	0.49062	0.42662	0.37964
		λ	0.46199	0.5876	8.00378	0.35147	0.2007	0.4968	0.38084	0.31159
	II	*γ*	0.31019	0.19043	0.21259	0.17659	0.17404	0.18807	0.1899	0.21173
		*θ*	1.01902	0.48466	0.75295	0.35237	0.26369	0.44573	0.38638	0.34024
		λ	0.3305	0.38752	1.16171	0.26717	0.16662	0.34442	0.2838	0.24285
	III	*γ*	0.41288	0.2389	0.27618	0.21289	0.187	0.23191	0.22513	0.23631
		*θ*	1.31423	0.57986	0.90941	0.41821	0.29104	0.53368	0.46076	0.39663
		λ	0.28608	0.32666	0.81081	0.24471	0.16978	0.29658	0.25582	0.2332
(30,24) (40,32)	I	*γ*	0.34704	0.21758	0.2392	0.20417	0.20236	0.21646	0.22096	0.24757
		*θ*	1.05666	0.49599	0.75756	0.36728	0.28438	0.45981	0.4052	0.36466
		λ	0.30532	0.36678	2.14913	0.25358	0.16019	0.32238	0.26234	0.22527
	II	*γ*	0.30591	0.19337	0.2159	0.17831	0.16888	0.19016	0.18939	0.20466
		*θ*	1.02283	0.50042	0.76075	0.36829	0.27011	0.46201	0.40241	0.35308
		λ	0.26702	0.30988	0.583	0.23595	0.16283	0.28194	0.24322	0.21982
	III	*γ*	0.29752	0.18261	0.20724	0.16633	0.15703	0.17855	0.17649	0.19135
		*θ*	1.01499	0.48854	0.75485	0.35123	0.24721	0.44756	0.38344	0.32903
		λ	0.27749	0.31888	0.57399	0.23971	0.15751	0.28882	0.2455	0.21444
(60,36) (70,42)	I	*γ*	0.20181	0.14888	0.15949	0.14219	0.14175	0.14832	0.1505	0.16306
		*θ*	0.54044	0.36364	0.47318	0.29702	0.2309	0.34504	0.31544	0.28897
		λ	0.1837	0.20506	0.27168	0.16864	0.12435	0.19056	0.16972	0.15625
	II	*γ*	0.1766	0.13397	0.14239	0.1282	0.12487	0.13315	0.13375	0.14081
		*θ*	0.50255	0.34381	0.43598	0.28574	0.22266	0.32799	0.30235	0.27828
		λ	0.12162	0.13227	0.15947	0.11549	0.09358	0.12536	0.11569	0.11046
	III	*γ*	0.18261	0.13738	0.148	0.12982	0.12297	0.13568	0.13467	0.13972
		*θ*	0.47765	0.32941	0.41936	0.27094	0.20348	0.31272	0.28525	0.2583
		λ	0.11733	0.12698	0.15588	0.11111	0.08923	0.12041	0.11094	0.10494
(60,48) (70,56)	I	*γ*	0.17093	0.13003	0.13771	0.12498	0.12362	0.12952	0.13077	0.13885
		*θ*	0.48204	0.33226	0.42199	0.27529	0.21363	0.31629	0.29047	0.26633
		λ	0.13787	0.15141	0.1872	0.13036	0.10153	0.14295	0.1305	0.12168
	II	*γ*	0.15313	0.11945	0.12628	0.11482	0.11256	0.11883	0.11944	0.12543
		*θ*	0.40923	0.28908	0.3626	0.242	0.19219	0.27589	0.25472	0.23551
		λ	0.12945	0.14071	0.16704	0.12287	0.09535	0.13325	0.12175	0.11223
	III	*γ*	0.1439	0.11066	0.11591	0.10768	0.11074	0.11084	0.11325	0.12248
		*θ*	0.41668	0.28684	0.35556	0.24376	0.20278	0.27548	0.25777	0.24326
		λ	0.11755	0.12696	0.1487	0.11236	0.09093	0.12103	0.11232	0.10647

**Table 3:** RABs for the MLEs and Bayes estimates of *γ*, *θ*, λ for different schemes with *T*_1_ = 0.9, *T*_2_ = 0.5. Prior parameter values are: *μ* = 2, *ν* = 1.5, *ζ* = 1.5, *η* = 1.5. Population parameter values are: *γ* = 1.85699, *θ* = 2.50899, λ = 1.5.

(*n*_2_, *m*_2_)	CS	$\hat{\alpha }$	ML	Bayes						
				SE	LINEX			GE		
					*c* = −0.5	*c* = 0.5	*c* = 2.0	*c* = −0.5	*c* = 0.5	*c* = 2.0
(30,18) (40,24)	I	*γ*	0.20275	0.11854	0.1349	0.10885	0.10921	0.11715	0.11946	0.13832
		*θ*	0.47521	0.21257	0.34179	0.15362	0.11845	0.19555	0.17004	0.15131
		λ	0.30799	0.39174	5.33585	0.23431	0.1338	0.3312	0.25389	0.20773
	II	*γ*	0.16704	0.10255	0.11448	0.0951	0.09372	0.10128	0.10226	0.11402
		*θ*	0.40615	0.19317	0.3001	0.14044	0.1051	0.17765	0.154	0.13561
		λ	0.22033	0.25835	0.77447	0.17811	0.11108	0.22962	0.1892	0.1619
	III	*γ*	0.22234	0.12865	0.14873	0.11464	0.1007	0.12489	0.12123	0.12725
		*θ*	0.52381	0.23111	0.36246	0.16669	0.116	0.21271	0.18364	0.15808
		λ	0.19072	0.21777	0.54054	0.16314	0.11319	0.19772	0.17055	0.15547
(30,24) (40,32)	I	*γ*	0.18688	0.11717	0.12881	0.10995	0.10897	0.11656	0.11899	0.13332
		*θ*	0.42115	0.19769	0.30194	0.14639	0.11335	0.18327	0.1615	0.14534
		λ	0.20354	0.24452	1.43275	0.16905	0.1068	0.21492	0.1749	0.15018
	II	*γ*	0.16474	0.10413	0.11626	0.09602	0.09094	0.1024	0.10199	0.11021
		*θ*	0.40767	0.19945	0.30321	0.14679	0.10766	0.18414	0.16039	0.14073
		λ	0.17802	0.20659	0.38867	0.1573	0.10855	0.18796	0.16215	0.14655
	III	*γ*	0.16022	0.09834	0.1116	0.08957	0.08456	0.09615	0.09504	0.10305
		*θ*	0.40454	0.19471	0.30086	0.13999	0.09853	0.17838	0.15283	0.13114
		λ	0.185	0.21259	0.38266	0.15981	0.10501	0.19255	0.16367	0.14296
(60,36) (70,42)	I	*γ*	0.10867	0.08017	0.08588	0.07657	0.07633	0.07987	0.08105	0.08781
		*θ*	0.2154	0.14494	0.1886	0.11838	0.09203	0.13752	0.12572	0.11518
		λ	0.12246	0.13671	0.18112	0.11243	0.0829	0.12704	0.11314	0.10416
	II	*γ*	0.0951	0.07214	0.07668	0.06904	0.06724	0.0717	0.07202	0.07583
		*θ*	0.2003	0.13703	0.17377	0.11388	0.08875	0.13073	0.12051	0.11091
		λ	0.08108	0.08818	0.10631	0.07699	0.06239	0.08358	0.07713	0.07364
	III	*γ*	0.09834	0.07398	0.0797	0.06991	0.06622	0.07307	0.07252	0.07524
		*θ*	0.19038	0.13129	0.16714	0.10799	0.0811	0.12464	0.11369	0.10295
		λ	0.07822	0.08465	0.10392	0.07407	0.05948	0.08028	0.07396	0.06996
(60,48) (70,56)	I	*γ*	0.09205	0.07002	0.07416	0.0673	0.06657	0.06975	0.07042	0.07477
		*θ*	0.19213	0.13243	0.16819	0.10972	0.08514	0.12606	0.11577	0.10615
		λ	0.09191	0.10094	0.1248	0.08691	0.06769	0.0953	0.087	0.08112
	II	*γ*	0.08246	0.06432	0.068	0.06183	0.06061	0.06399	0.06432	0.06754
		*θ*	0.1631	0.11522	0.14452	0.09645	0.0766	0.10996	0.10152	0.09386
		λ	0.0863	0.09381	0.11136	0.08191	0.06356	0.08883	0.08116	0.07482
	III	*γ*	0.07749	0.05959	0.06242	0.05799	0.05963	0.05969	0.06099	0.06595
		*θ*	0.16607	0.11433	0.14172	0.09715	0.08082	0.1098	0.10274	0.09696
		λ	0.07836	0.08464	0.09914	0.07491	0.06062	0.08068	0.07488	0.07098

**Table 4:** AILs and CPs of 95% CIs of *γ*, *θ*, λ for different schemes with *T*_1_ = 0.9, *T*_2_ = 0.5. Prior parameter values are: *μ* = 2, *ν* = 1.5, *ζ* = 1.5, *η* = 1.5. Population parameter values are: *γ* = 1.85699, *θ* = 2.50899, λ = 1.5.

(*n*_1_, *m*_1_)	CS	*α*	NACI	LTCI	Credible interval
			AIL(CP)	AIL(CP)	AIL(CP)
(30,18) (40,24)	I	*γ*	2.40472(0.949)	2.57646(0.938)	2.06628(0.972)
		*θ*	3.72638(0.948)	4.01016(0.953)	3.03154(0.967)
		λ	2.43485(0.952)	2.69729(0.965)	2.76214(0.958)
	II	*γ*	2.16746(0.943)	2.28778(0.94)	1.89708(0.967)
		*θ*	3.52143(0.965)	3.75672(0.946)	2.92181(0.97)
		λ	2.0486(0.958)	2.20408(0.969)	2.27354(0.966)
	III	*γ*	2.29102(0.941)	2.42344(0.924)	1.98566(0.968)
		*θ*	3.63263(0.956)	3.87924(0.934)	2.96345(0.961)
		λ	1.93216(0.934)	2.07115(0.961)	2.1465(0.956)
(30,24) (40,32)	I	*γ*	2.1537(0.937)	2.2768(0.926)	1.88806(0.957)
		*θ*	3.4642(0.97)	3.69375(0.945)	2.88556(0.971)
		λ	2.08114(0.968)	2.24647(0.968)	2.31096(0.962)
	II	*γ*	2.08385(0.935)	2.18832(0.93)	1.84099(0.96)
		*θ*	3.42006(0.959)	3.634(0.948)	2.86244(0.971)
		λ	1.88798(0.957)	2.01294(0.955)	2.09107(0.955)
	III	*γ*	2.14779(0.963)	2.25897(0.938)	1.88271(0.98)
		*θ*	3.49995(0.978)	3.72258(0.955)	2.9114(0.978)
		λ	1.89774(0.959)	2.01932(0.956)	2.10341(0.955)
(60,36) (70,42)	I	*γ*	1.73452(0.949)	1.79813(0.938)	1.59593(0.957)
		*θ*	2.57972(0.955)	2.68008(0.944)	2.33382(0.949)
		λ	1.66283(0.958)	1.74694(0.97)	1.77861(0.961)
	II	*γ*	1.55884(0.936)	1.60394(0.935)	1.45128(0.954)
		*θ*	2.38412(0.956)	2.46401(0.939)	2.17962(0.952)
		λ	1.41641(0.966)	1.46955(0.964)	1.50376(0.961)
	III	*γ*	1.60071(0.943)	1.64814(0.931)	1.4849(0.95)
		*θ*	2.39912(0.964)	2.47834(0.95)	2.19064(0.965)
		λ	1.37189(0.964)	1.41944(0.966)	1.45824(0.965)
(60,48) (70,56)	I	*γ*	1.55716(0.95)	1.60237(0.933)	1.44975(0.96)
		*θ*	2.39881(0.954)	2.47953(0.934)	2.19836(0.954)
		λ	1.43074(0.967)	1.48433(0.964)	1.51912(0.959)
	II	*γ*	1.48253(0.944)	1.52127(0.939)	1.38941(0.955)
		*θ*	2.28809(0.96)	2.35919(0.949)	2.11081(0.965)
		λ	1.36095(0.964)	1.40605(0.953)	1.44217(0.953)
	III	*γ*	1.50142(0.96)	1.54259(0.949)	1.40172(0.967)
		*θ*	2.25812(0.958)	2.32824(0.948)	2.0774(0.968)
		λ	1.34054(0.97)	1.3845(0.957)	1.42105(0.959)

**Table 5:** MSEs for the MLEs and Bayes estimates of *γ*, *θ*, λ for different schemes with *T*_1_ = 0.9, *T*_2_ = 0.5. Prior parameter values are: *μ* = 2, *ν* = 1.5, *ζ* = 1.5, *η* = 1.5. Population parameter values are: *γ* = 1.85699, *θ* = 2.50899, λ = 1.5.

(*n*_2_, *m*_2_)	CS	$\hat{\alpha }$	ML	Bayes						
				SE	LINEX			GE		
					*c* = −0.5	*c* = 0.5	*c* = 2.0	*c* = −0.5	*c* = 0.5	*c* = 2.0
(30,18) (30,18)	I	*γ*	0.50423	0.26933	0.31365	0.24037	0.22127	0.26279	0.26055	0.29071
		*θ*	1.69432	0.65463	1.12204	0.4424	0.30002	0.5915	0.49394	0.41319
		λ	0.49618	0.66563	11.5727	0.36847	0.20848	0.54655	0.40302	0.33925
	II	*γ*	0.34926	0.19796	0.22127	0.18409	0.18628	0.19627	0.20063	0.23064
		*θ*	1.34928	0.56248	0.93061	0.39364	0.28483	0.51307	0.43744	0.37753
		λ	0.35297	0.44562	2.94538	0.29777	0.17558	0.38608	0.30605	0.2569
	III	*γ*	0.4545	0.24156	0.28323	0.21317	0.18846	0.23378	0.22672	0.2419
		*θ*	1.574	0.60486	1.01265	0.41596	0.28743	0.54949	0.4635	0.39164
		λ	0.30397	0.36612	1.28787	0.26259	0.17497	0.3255	0.27242	0.24741
(30,24) (30,24)	I	*γ*	0.3573	0.20931	0.23222	0.19594	0.19952	0.20841	0.21462	0.24832
		*θ*	1.16933	0.51063	0.81279	0.37553	0.30998	0.47206	0.41625	0.38132
		λ	0.31329	0.39195	1.83564	0.27158	0.17877	0.34355	0.28225	0.25752
	II	*γ*	0.33818	0.20165	0.22136	0.18996	0.19269	0.20099	0.20651	0.23531
		*θ*	1.23814	0.53646	0.8545	0.38852	0.2985	0.49433	0.43086	0.38378
		λ	0.31555	0.38484	1.29127	0.26187	0.16324	0.33806	0.27457	0.2367
	III	*γ*	0.35624	0.2026	0.22908	0.1856	0.17914	0.19916	0.19955	0.2219
		*θ*	1.28021	0.53433	0.86474	0.3783	0.27908	0.48854	0.41871	0.36438
		λ	0.27387	0.32799	0.7243	0.23721	0.15542	0.29186	0.24322	0.21788
(60,36) (60,36)	I	*γ*	0.21531	0.1558	0.16648	0.14939	0.1515	0.15571	0.15926	0.17513
		*θ*	0.56112	0.36552	0.48344	0.29636	0.23534	0.34600	0.31558	0.29025
		λ	0.18717	0.21005	0.28251	0.17237	0.13015	0.19554	0.17587	0.16686
	II	*γ*	0.15873	0.11937	0.12517	0.11633	0.12146	0.11988	0.1234	0.13567
		*θ*	0.45458	0.31022	0.39541	0.25934	0.21678	0.29607	0.27438	0.25759
		λ	0.15265	0.16867	0.21086	0.14374	0.1104	0.15858	0.14386	0.13392
	III	*γ*	0.17897	0.12991	0.14096	0.12222	0.11649	0.12814	0.1272	0.13309
		*θ*	0.52365	0.34081	0.44899	0.27267	0.19706	0.32112	0.2887	0.25674
		λ	0.13066	0.14069	0.16691	0.12344	0.09939	0.13367	0.12363	0.11774
(60,48) (60,48)	I	*γ*	0.16803	0.1253	0.13354	0.11994	0.1188	0.12461	0.12567	0.13408
		*θ*	0.492	0.31915	0.41444	0.26162	0.20771	0.30344	0.27865	0.25735
		λ	0.13607	0.14965	0.18458	0.12931	0.10295	0.14158	0.13033	0.12437
	II	*γ*	0.17679	0.13515	0.14291	0.12986	0.12696	0.13454	0.1354	0.14245
		*θ*	0.46446	0.31609	0.39756	0.26503	0.21432	0.30206	0.2799	0.26077
		λ	0.12665	0.13766	0.16415	0.12118	0.09962	0.13132	0.12255	0.11825
	III	*γ*	0.19202	0.14331	0.15356	0.13584	0.12809	0.14171	0.14072	0.14532
		*θ*	0.54092	0.36499	0.46938	0.29898	0.22319	0.34664	0.31637	0.28638
		λ	0.12797	0.1382	0.16219	0.12287	0.10216	0.13231	0.12416	0.12016

**Table 6:** RABs for the MLEs and Bayes estimates of *γ*, *θ*, λ for different schemes with *T*_1_ = 0.9, *T*_2_ = 0.5. Prior parameter values are: *μ* = 2, *ν* = 1.5, *ζ* = 1.5, *η* = 1.5. Population parameter values are: *γ* = 1.85699, *θ* = 2.50899, λ = 1.5.

(*n*_2_, *m*_2_)	CS	$\hat{\alpha }$	ML	Bayes						
				SE	LINEX			GE		
					*c* = −0.5	*c* = 0.5	*c* = 2.0	*c* = −0.5	*c* = 0.5	*c* = 2.0
(30,18) (30,18)	I	*γ*	0.27153	0.14504	0.1689	0.12944	0.11916	0.14152	0.14031	0.15655
		*θ*	0.6753	0.26091	0.44721	0.17632	0.11958	0.23575	0.19687	0.16468
		λ	0.33079	0.44375	7.71513	0.24565	0.13899	0.36437	0.26868	0.22617
	II	*γ*	0.18808	0.1066	0.11915	0.09913	0.10031	0.10569	0.10804	0.1242
		*θ*	0.53778	0.22419	0.37091	0.15689	0.11352	0.20449	0.17435	0.15047
		λ	0.23532	0.29708	1.96358	0.19851	0.11705	0.25739	0.20404	0.17127
	III	*γ*	0.24475	0.13008	0.15252	0.11479	0.10148	0.12589	0.12209	0.13027
		*θ*	0.62734	0.24108	0.40361	0.16579	0.11456	0.21901	0.18474	0.15609
		λ	0.20265	0.24408	0.85858	0.17506	0.11664	0.217	0.18162	0.16494
(30,24) (30,24)	I	*γ*	0.19241	0.11272	0.12505	0.10552	0.10745	0.11223	0.11558	0.13372
		*θ*	0.46606	0.20352	0.32395	0.14967	0.12355	0.18815	0.1659	0.15198
		λ	0.20886	0.2613	1.22376	0.18106	0.11918	0.22903	0.18817	0.17168
	II	*γ*	0.18211	0.10859	0.1192	0.1023	0.10377	0.10824	0.11121	0.12671
		*θ*	0.49348	0.21382	0.34058	0.15485	0.11897	0.19702	0.17173	0.15296
		λ	0.21037	0.25656	0.86085	0.17458	0.10883	0.22537	0.18305	0.1578
	III	*γ*	0.19184	0.1091	0.12336	0.09995	0.09647	0.10725	0.10746	0.1195
		*θ*	0.51025	0.21297	0.34466	0.15078	0.11123	0.19472	0.16689	0.14523
		λ	0.18258	0.21866	0.48286	0.15814	0.10361	0.19457	0.16215	0.14525
(60,36) (60,36)	I	*γ*	0.11595	0.0839	0.08965	0.08045	0.08158	0.08385	0.08576	0.09431
		*θ*	0.22365	0.14569	0.19268	0.11812	0.0938	0.1379	0.12578	0.11568
		λ	0.12478	0.14004	0.18834	0.11491	0.08676	0.13036	0.11725	0.11124
	II	*γ*	0.08548	0.06428	0.06741	0.06264	0.06541	0.06456	0.06645	0.07306
		*θ*	0.18118	0.12364	0.1576	0.10336	0.0864	0.11801	0.10936	0.10267
		λ	0.10177	0.11244	0.14057	0.09583	0.0736	0.10572	0.09591	0.08928
	III	*γ*	0.09638	0.06996	0.07591	0.06582	0.06273	0.069	0.0685	0.07167
		*θ*	0.20871	0.13583	0.17895	0.10868	0.07854	0.12799	0.11507	0.10233
		λ	0.08711	0.0938	0.11128	0.08229	0.06626	0.08911	0.08242	0.07849
(60,48) (60,48)	I	*γ*	0.09049	0.06747	0.07191	0.06459	0.06397	0.0671	0.06767	0.0722
		*θ*	0.19609	0.1272	0.16518	0.10427	0.08278	0.12094	0.11106	0.10257
		λ	0.09072	0.09977	0.12305	0.08621	0.06863	0.09439	0.08689	0.08291
	II	*γ*	0.0952	0.07278	0.07696	0.06993	0.06837	0.07245	0.07291	0.07671
		*θ*	0.18512	0.12598	0.15845	0.10563	0.08542	0.12039	0.11156	0.10393
		λ	0.08444	0.09177	0.10943	0.08079	0.06641	0.08755	0.0817	0.07883
	III	*γ*	0.1034	0.07717	0.08269	0.07315	0.06898	0.07631	0.07578	0.07826
		*θ*	0.21559	0.14547	0.18708	0.11916	0.08896	0.13816	0.1261	0.11414
		λ	0.08531	0.09213	0.10813	0.08191	0.0681	0.08821	0.08277	0.08011

**Table 7:** AILs and CPs of 95% CIs of *γ*, *θ*, λ for different schemes with *T*_1_ = 0.9, *T*_2_ = 0.5. Prior parameter values are: *μ* = 2, *ν* = 1.5, *ζ* = 1.5, *η* = 1.5. Population parameter values are: *γ* = 1.85699, *θ* = 2.50899, λ = 1.5.

(*n*_1_, *m*_1_) (*n*_2_, *m*_2_)	CS	*α*	NACI	LTCI	Credible interval
			AIL(CP)	AIL(CP)	AIL(CP)
(30,18) (30,18)	I	*γ*	2.58857(0.935)	2.7907(0.913)	2.16533(0.962)
		*θ*	4.27887(0.962)	4.66559(0.939)	3.31008(0.976)
		λ	2.56928(0.967)	2.90091(0.972)	2.9757(0.967)
	II	*γ*	2.2876(0.945)	2.43009(0.937)	1.95846(0.98)
		*θ*	3.88053(0.967)	4.18178(0.948)	3.11216(0.977)
		λ	2.24882(0.97)	2.44813(0.961)	2.56041(0.961)
	III	*γ*	2.44597(0.947)	2.6032(0.931)	2.06634(0.971)
		*θ*	4.03306(0.966)	4.35859(0.95)	3.16148(0.977)
		λ	2.0876(0.956)	2.25769(0.955)	2.37414(0.963)
(30,24) (30,24)	I	*γ*	2.27984(0.951)	2.42581(0.941)	1.96047(0.971)
		*θ*	3.7094(0.964)	3.99489(0.953)	3.01029(0.979)
		λ	2.20611(0.969)	2.41108(0.966)	2.49686(0.967)
	II	*γ*	2.18031(0.941)	2.30664(0.941)	1.88403(0.968)
		*θ*	3.68993(0.952)	3.96129(0.95)	2.99598(0.967)
		λ	2.11522(0.975)	2.29024(0.966)	2.38414(0.966)
	III	*γ*	2.27082(0.951)	2.40513(0.936)	1.95005(0.978)
		*θ*	3.79297(0.961)	4.07642(0.947)	3.05093(0.975)
		λ	2.05294(0.966)	2.21292(0.97)	2.3137(0.969)
(60,36) (60,36)	I	*γ*	1.78413(0.95)	1.8544(0.944)	1.63079(0.964)
		*θ*	2.67722(0.943)	2.78988(0.946)	2.40591(0.957)
		λ	1.71545(0.962)	1.8102(0.971)	1.84465(0.964)
	II	*γ*	1.59009(0.953)	1.63965(0.955)	1.47242(0.967)
		*θ*	2.44049(0.955)	2.52871(0.954)	2.22355(0.965)
		λ	1.51153(0.968)	1.57427(0.963)	1.6127(0.952)
	III	*γ*	1.65601(0.954)	1.70825(0.947)	1.52539(0.97)
		*θ*	2.53399(0.964)	2.62557(0.95)	2.28974(0.966)
		λ	1.42168(0.958)	1.47437(0.959)	1.51675(0.961)
(60,48) (60,48)	I	*γ*	1.60188(0.945)	1.65089(0.953)	1.48044(0.964)
		*θ*	2.46568(0.955)	2.55437(0.951)	2.23929(0.958)
		λ	1.47532(0.97)	1.53486(0.966)	1.56959(0.972)
	II	*γ*	1.52941(0.935)	1.57207(0.93)	1.4247(0.945)
		*θ*	2.37418(0.954)	2.45397(0.945)	2.17144(0.949)
		λ	1.38675(0.954)	1.43731(0.966)	1.47283(0.966)
	III	*γ*	1.57091(0.919)	1.61586(0.915)	1.45886(0.938)
		*θ*	2.43516(0.95)	2.51801(0.932)	2.21839(0.944)
		λ	1.36062(0.948)	1.40863(0.948)	1.44806(0.949)

### 5.1 Simulation results

Based on the numerical calculations presented in Tables 2–7, we can observe the following points:

1. The MSEs and RABs decrease as the value of sample size increases for all cases.
2. For fixed values of the sample size, by increasing the observed failure times the MSEs and RABs of the underlying parameters decrease.
3. The results presented in Tables 2 and 3 are based on different sample sizes (*n*_1_ < *n*_2_) while those presented in Tables 5 and 6 are based on the same sample sizes (*n*_1_ = *n*_2_). Better results are obtained when considering *n*_1_ < *n*_2_. This can be seen when we compare the results presented in Tables 2 and 3 with those in Tables 5 and 6. This is due to a greater number of units subject to acceleration will have smaller lifetimes (and hence smaller MSEs) than those under normal stress conditions.
4. The Bayes estimates under the LINEX loss function (*c* = 2) have the smallest MSEs and RABs as compared with the Bayes estimates under SE, GE loss functions, and MLEs.
5. For fixed values *n*_*j*_, *m*_*j*_, *j* = 1, 2, better results (through comparing the MSEs and RABs) are obtained under progressive CS III than those obtained under progressive CSs I and II.
6. For fixed values of the sample size, by increasing the observed failure times, the AILs for the credible intervals are smaller than those for the NACIs and LTCIs. Also, the CPs of the credible intervals are closer to 95% than those for NACIs and LTCIs.
7. The AILs of the NACIs, LTCIs and credible intervals decrease as the sample size increases.

## 6 Real data analysis

**Fig 3:**
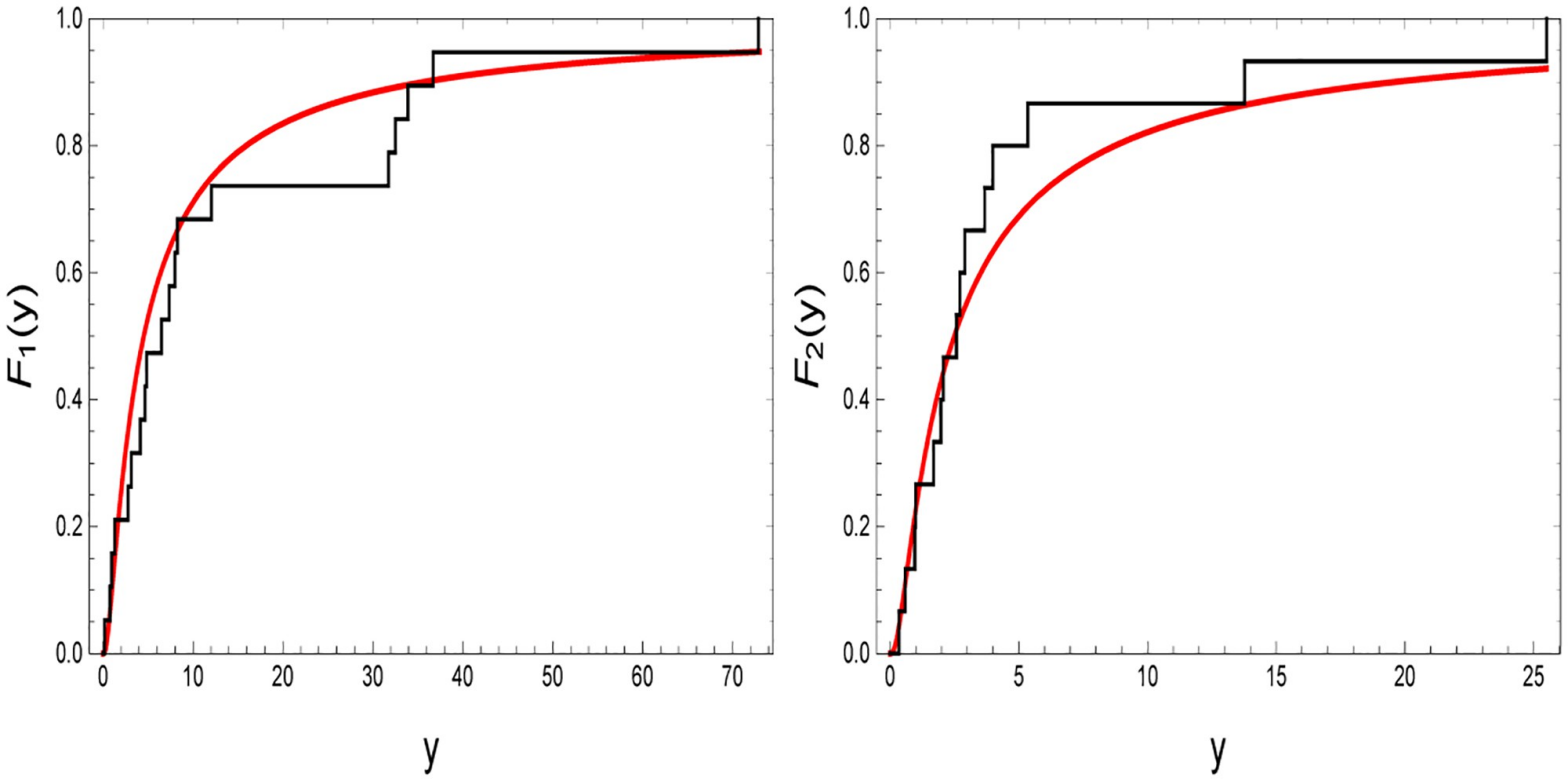
Empirical CDFs against CDFs of the IKum distribution for the given data listed in Table 8, under two the stress levels.

In this section, a real data set is introduced to show how the ML and Bayes estimation methods work in practice based on real data from Nelson [1]. The data are presented in Table 8. They correspond to the oil breakdown times of insulating fluid under two stress levels (34 kilovolt (kv) and 36 kv), considering the data set under 34 kv as data under normal stress. Before further proceeding, we test the validity of IKum distribution to fit the data listed in Table 8 using Kolmogorov-Smirnov (K-S) test statistic and its corresponding *p*-value for each stress level. The results are listed in Table 9 in which we can notice that the IKum distribution fits the given data, under the two stress levels, well because the *p*-values are greater than 0.05. This is done graphically by plotting the empirical CDFs of the two data sets against the CDFs of the IKum distribution, see the two panels in Fig 3.

**Table 8:** Times to breakdown of an insulating fluid.

Stress	Complete failure data
34 kv	0.19, 0.78, 0.96, 1.31, 2.78, 3.16, 4.15, 4.67, 4.85, 6.50, 7.35, 8.01, 8.27, 12.06, 31.75, 32.52, 33.91, 36.71, 72.89
36 kv	0.35, 0.59, 0.96, 0.99, 1.69, 1.97, 2.07, 2.58, 2.71, 2.9, 3.67, 3.99, 5.35, 13.77, 25.5

**Table 9:** MLEs of the parameters, K-S statistic and *p*-value.

Parameters	Stress	K-S	*p*-value
*γ* = 0.951196, *θ* = 3.16839, λ = 1.82019	34 kv	0.104776	0.985184
	36 kv	0.166469	0.800124

We apply Type-I PHCS to the real data set listed in Table 8 to obtain three samples subjecting to Type-I PHCS see Table 10, where the three Type-I PHCSs are considered as follows: We assume that *n*_1_ = 19, *m*_1_ = 15, *T*_1_ = 34, *n*_2_ = 15, *m*_2_ = 12, *T*_2_ = 12 and

**CS I:** *R*_1*i*_ = (4, 14 * 0) and *R*_2*i*_ = (3, 11 * 0).
**CS II:** *R*_1*i*_ = (14 * 0, 4) and *R*_2*i*_ = (11 * 0, 3).
**CS III:** *R*_1*i*_ = (1, 1, 1, 1, 11 * 0) and *R*_2*i*_ = (1, 1, 1, 9 * 0).

**Table 10:** The three samples under Type-I PHCSs.

CS	Stress	Censored failure data
I	34 kv	0.19, 3.16, 4.15, 4.67, 4.85, 6.50, 7.35, 8.01, 8.27, 12.06, 31.75, 32.52, 33.91
	36 kv	0.35, 1.69, 1.97, 2.07, 2.58, 2.71, 2.9, 3.67, 3.99, 5.35
II	34 kv	0.19, 0.78, 0.96, 1.31, 2.78, 3.16, 4.15, 4.67, 4.85, 6.50, 7.35, 8.01, 8.27, 12.06, 31.75
	36 kv	0.35, 0.59, 0.96, 0.99, 1.69, 1.97, 2.07, 2.58, 2.71, 2.9, 3.67, 3.99
III	34 kv	0.19, 0.96, 2.78, 4.15, 4.85, 6.50, 7.35, 8.01, 8.27, 12.06, 31.75, 32.52, 33.91
	36 kv	0.35, 0.96, 1.69, 2.07, 2.58, 2.71, 2.9, 3.67, 3.99, 5.35

Based on the three samples shown in Table 10, which are subject to Type-I PHCS, the ML and Bayes estimates of the parameters *γ*, *θ*, λ are calculated and presented in Table 11, while the 95% confidence and credible intervals are presented in Table 12. In addition, the mean time to failure (MTTF) and RF ($R_{1}\left(y\right)=1-F_{1}\left(y\right)$) for certain mission times are calculated and given in Table 11 under normal operating conditions.

**Table 11:** MLEs and Bayes estimates of (*γ*, *θ*, λ), MTTF and RF based on the three samples given in Table 10.

CS		ML	Bayes						
			SE	LINEX			GE		
				*c* = −0.5	*c* = 0.5	*c* = 2.0	*c* = −0.5	*c* = 0.5	*c* = 2.0
I	*γ*	0.80607	0.76062	0.76687	0.75444	0.73638	0.75234	0.73533	0.70849
	*θ*	3.4368	3.28418	3.67977	3.00461	2.46949	3.18952	3.00311	2.72753
	λ	1.93185	2.33276	3.35438	1.99637	1.53498	2.16624	1.86995	1.49319
	MTTF	31.4606	35.6192	72.867	18.8448	9.45241	34.5295	32.2951	28.841
	$R_{1}\left(1\right)$	0.94585	0.92865	0.92922	0.92807	0.92624	0.92800	0.92666	0.92450
	$R_{1}\left(5\right)$	0.60337	0.60374	0.60594	0.60152	0.59480	0.59994	0.59199	0.57903
	$R_{1}\left(10\right)$	0.41568	0.42907	0.43127	0.42688	0.42034	0.42379	0.41271	0.39442
II	*γ*	0.83225	0.78102	0.78846	0.77366	0.75208	0.77134	0.7513	0.7193
	*θ*	2.76564	2.61204	2.81307	2.44973	2.098	2.5454	2.41255	2.21623
	λ	1.95476	2.26595	2.67314	2.01231	1.59568	2.14124	1.9056	1.59107
	MTTF	24.0693	28.0631	57.9538	15.7605	8.84517	27.0606	25.0296	22.0067
	$R_{1}\left(1\right)$	0.89781	0.87998	0.88082	0.87913	0.87647	0.87898	0.87691	0.87360
	$R_{1}\left(5\right)$	0.50605	0.51134	0.51321	0.50946	0.50378	0.50753	0.49953	0.48639
	$R_{1}\left(10\right)$	0.33239	0.34880	0.35054	0.34707	0.34192	0.34366	0.33287	0.31507
III	*γ*	0.76109	0.72478	0.73044	0.71919	0.7029	0.71694	0.70091	0.67564
	*θ*	3.06307	2.95534	3.21135	2.75989	2.3697	2.88397	2.74533	2.54861
	λ	1.86757	2.25527	2.83812	1.94512	1.50242	2.09915	1.81549	1.45605
	MTTF	33.0938	36.7329	70.0858	19.9137	12.524	35.6813	33.5215	30.1914
	$R_{1}\left(1\right)$	0.93487	0.92038	0.92090	0.91984	0.91819	0.91978	0.91856	0.91664
	$R_{1}\left(5\right)$	0.59530	0.59637	0.59834	0.59439	0.58840	0.59295	0.58585	0.57450
	$R_{1}\left(10\right)$	0.41637	0.42825	0.43029	0.42622	0.42016	0.42336	0.41319	0.39685

**Table 12:** NACIs, LTCIs and credible intervals of the parameters based on the three samples given in Table 10.

CS	*α*	NACI	LTCI	Credible interval
I	*γ*	(0.48658, 1.12557)	(0.54230, 1.19816)	(0.46338, 1.07602)
	*θ*	(1.14280, 5.73079)	(1.76307, 6.69942)	(1.53640, 6.00954)
	λ	(0.00000, 3.88226)	(0.70390, 5.3020)	(0.67655, 5.8207)
II	*γ*	(0.49401, 1.17049)	(0.55430, 1.24956)	(0.45864, 1.13261)
	*θ*	(1.07735, 4.45394)	(1.50203, 5.09228)	(1.24381, 4.52290)
	λ	(0.18940, 3.72012)	(0.79228, 4.82290)	(0.75535, 5.12422)
III	*γ*	(0.44552, 1.07667)	(0.50276, 1.15216)	(0.4539, 1.04092)
	*θ*	(1.11107, 5.01507)	(1.61955, 5.79320)	(1.54113, 5.17004)
	λ	(0.00000, 3.73687)	(0.68640, 5.08129)	(0.68557, 5.72437)

## 7 Conclusion

When the lifetimes of items under use conditions are subject to the IKum distribution, we have addressed the issue of point and interval estimations under constant-stress PALT. To estimate the model parameters, the ML and Bayes (under LINEX, GE, and SE loss functions) methods have been applied based on Type-I PHCS. In addition, the estimated CIs have been acquired as well as credible intervals. To get Bayes estimates of the model parameters, the MCMC technique has been implemented. To investigate the accuracy of the estimates obtained and to compare the output of the CIs, a simulation study has been developed. To discuss and test the efficiency of the suggested estimation methods, a real data set has been considered. The actual data results show that the IKum distribution is a good candidate for fitting the data and the methods of estimation perform well under Type-I PHCS as well. Finally, we recommend the use of Bayes estimation technique under LINEX loss function with values of *c* closer to 2 to estimate the model parameters under consideration.

It should be noted that if the hyperparameters are unknown, we can use the empirical Bayes method to estimate them using past samples, see [43]. Instead, one may use the hierarchical Bayes approach in which suitable priors for the hyperparameters could be used, see [44].

**Future work:** Neutrosophic statistics is the extension of classical statistics and is applied when the data are coming from a complex process or an uncertain environment. So, the current study can be extended using neutrosophic statistics as future research, see [45–47].

## Supporting information

S1 Appendix (PDF)
