# Supplementary material for: Statistical inference for a constant-stress partially accelerated life tests based on progressively hybrid censored samples from inverted Kumaraswamy distribution
Source: PLoS One. 2022 Aug 1;17(8):e0272378. doi: 10.1371/journal.pone.0272378 (PMC9342795; doi:10.1371/journal.pone.0272378)
Supplement: S1 Appendix — (PDF) [file pone.0272378.s001.pdf]

Appendix A The second partial derivatives of logarithm of LF for γ, θ and λ are given by

$$
\begin{aligned}
\frac{\partial^2 l}{\partial \gamma^2} =& -\frac{D_1 + D_2}{\gamma^2} - \sum_{j=1}^{2} \Bigg[\sum_{i=1}^{D_j} \Bigg((1-\theta)\psi_1(\lambda^{j-1}, y_{ji:m_j:n_j}) + \theta R_{ji} G_{2j}(\lambda^{j-1}, y_{ji:m_j:n_j}) \\
& \times \psi_2(\lambda^{j-1}, y_{ji:m_j:n_j}) \Bigg) + \theta R^*_{D_j} G_{2j}(\lambda^{j-1}, T_j) \psi_2(\lambda^{j-1}, T_j) \Bigg], \\
\frac{\partial^2 l}{\partial \theta^2} =& -\frac{D_1 + D_2}{\theta^2} - \sum_{j=1}^{2} \Bigg[\sum_{i=1}^{D_j} \Bigg(R_{ji} \frac{[G_{2j}(\lambda^{j-1}, y_{ji:m_j:n_j})]^2}{[G_{1j}(\lambda^{j-1}, y_{ji:m_j:n_j})]^\theta} [\ln G_{1j}(\lambda^{j-1}, y_{ji:m_j:n_j})]^2 \Bigg) \\
& + R^*_{D_j} \frac{[G_{2j}(\lambda^{j-1}, T_j)]^2}{[G_{1j}(\lambda^{j-1}, T_j)]^\theta} [\ln G_{1j}(\lambda^{j-1}, T_j)]^2 \Bigg],
\end{aligned}
$$

$$
\begin{aligned}
\frac{\partial^2 l}{\partial \theta \partial \gamma} =& \sum_{j=1}^{2} \Bigg[\sum_{i=1}^{D_j} \Bigg(\frac{1}{G_{1j}(\lambda^{j-1}, y_{ji:m_j:n_j})} \frac{\partial G_{1j}(\lambda^{j-1}, y_{ji:m_j:n_j})}{\partial \gamma} \Bigg[1 - R_{ji} G_{2j}(\lambda^{j-1}, y_{ji:m_j:n_j}) \\
& \times \Bigg\{ 1 + \theta \frac{G_{2j}(\lambda^{j-1}, y_{ji:m_j:n_j})}{[G_{1j}(\lambda^{j-1}, y_{ji:m_j:n_j})]^\theta} \ln G_{1j}(\lambda^{j-1}, y_{ji:m_j:n_j}) \Bigg\} \Bigg] \Bigg) - R^*_{D_j} \frac{G_{2j}(\lambda^{j-1}, T_j)}{G_{1j}(\lambda^{j-1}, T_j)} \\
& \times \frac{\partial G_{1j}(\lambda^{j-1}, T_j)}{\partial \gamma} \Bigg\{ 1 + \theta \frac{G_{2j}(\lambda^{j-1}, T_j)}{[G_{1j}(\lambda^{j-1}, T_j)]^\theta} \ln G_{1j}(\lambda^{j-1}, T_j) \Bigg\} \Bigg], \\
\frac{\partial^2 l}{\partial \lambda^2} =& -\frac{D_2}{\lambda^2} - \sum_{i=1}^{D_2} \Bigg(-\frac{(\gamma+1) y^2_{2i:m_2:n_2}}{[G_2(\lambda, y_{2i:m_2:n_2})]^2} + (1-\theta)\psi_3(\lambda, y_{2i:m_2:n_2}) \\
& + \theta R_{2i} G_{22}(\lambda, y_{2i:m_2:n_2}) \psi_4(\lambda, y_{2i:m_2:n_2}) \Bigg) - \theta R^*_{D_2} G_{22}(\lambda, T_2) \psi_4(\lambda, T_2),
\end{aligned}
$$

$$
\begin{aligned}
\frac{\partial^2 l}{\partial \lambda \partial \gamma} =& -\sum_{i=1}^{D_2} \Bigg(\frac{y_{2i:m_2:n_2}}{G_2(\lambda, y_{2i:m_2:n_2})} + (1-\theta)\psi_5(\lambda, y_{2i:m_2:n_2}) + \theta R_{2i} G_{22}(\lambda, y_{2i:m_2:n_2}) \\
& \times \psi_6(\lambda, y_{2i:m_2:n_2}) \Bigg) - \theta R^*_{D_2} G_{22}(\lambda, T_2) \psi_6(\lambda, T_2), \\
\frac{\partial^2 l}{\partial \lambda \partial \theta} =& \sum_{i=1}^{D_2} \Bigg(\frac{1}{G_{12}(\lambda, y_{2i:m_2:n_2})} \frac{\partial G_{12}(\lambda, y_{2i:m_2:n_2})}{\partial \lambda} \Bigg[1 - R_{2i} G_{22}(\lambda, y_{2i:m_2:n_2}) \\
& \times \Bigg\{ 1 + \theta \frac{G_{22}(\lambda, y_{2i:m_2:n_2})}{[G_{12}(\lambda, y_{ji:m_j:n_j})]^\theta} \ln G_{12}(\lambda, y_{2i:m_2:n_2}) \Bigg\} \Bigg] \Bigg) - R^*_{D_2} \frac{G_{22}(\lambda, T_2)}{G_{12}(\lambda, T_2)} \\
& \frac{\partial G_{12}(\lambda, T_2)}{\partial \lambda} \Bigg\{ 1 + \theta \frac{G_{22}(\lambda, T_2)}{[G_{12}(\lambda, T_2)]^\theta} \ln G_{12}(\lambda, T_2) \Bigg\},
\end{aligned}
$$

where

$$\psi_1(\lambda^{j-1}, y_{ji:m_j:n_j}) = \left(\frac{1 + [G_j(\lambda^{j-1}, y_{ji:m_j:n_j})]^{\gamma} G_{1j}(\lambda^{j-1}, y_{ji:m_j:n_j})}{[G_{1j}(\lambda^{j-1}, y_{ji:m_j:n_j})]^2} \right) \times \left(\frac{\partial G_{1j}(\lambda^{j-1}, y_{ji:m_j:n_j})}{\partial \gamma} \right)^2,$$

$$\psi_2(\lambda^{j-1}, y_{ji:m_j:n_j}) = \frac{\theta}{[G_{1j}(\lambda^{j-1}, y_{ji:m_j:n_j})]^2} \left(\frac{\partial G_{1j}(\lambda^{j-1}, y_{ji:m_j:n_j})}{\partial \gamma} \right)^2 - \psi_1(\lambda^{j-1}, y_{ji:m_j:n_j}),$$

$$\psi_3(\lambda, y_{2i:m_2:n_2}) = \frac{-1}{G_{12}(\lambda, y_{2i:m_2:n_2})} \left(1 + \frac{1+\gamma}{\gamma} G_{12}(\lambda, y_{2i:m_2:n_2}) [G_2(\lambda, y_{2i:m_2:n_2})]^{\gamma} \right) \times \left(\frac{\partial G_{12}(\lambda, y_{2i:m_2:n_2})}{\partial \lambda} \right)^2,$$

$$\psi_4(\lambda, y_{2i:m_2:n_2}) = \frac{\theta G_{22}(\lambda, y_{ji:m_j:n_j})}{[G_{12}(\lambda, y_{2i:m_2:n_2})]^{\theta+2}} \left(\frac{\partial G_{12}(\lambda, y_{2i:m_2:n_2})}{\partial \lambda} \right)^2 + \psi_3(\lambda, y_{2i:m_2:n_2}),$$

$$\psi_5(\lambda, y_{2i:m_2:n_2}) = \frac{1}{G_{12}(\lambda, y_{2i:m_2:n_2})} \left(\frac{1}{\gamma} - \ln G_2(\lambda, y_{2i:m_2:n_2}) \left[1 + \frac{[G_2(\lambda, y_{2i:m_2:n_2})]^{-\gamma}}{G_{12}(\lambda, y_{2i:m_2:n_2})} \right] \right) \times \frac{\partial G_{12}(\lambda, y_{2i:m_2:n_2})}{\partial \lambda},$$

$$\psi_6(\lambda, y_{2i:m_2:n_2}) = \frac{\theta G_{22}(\lambda, y_{ji:m_j:n_j})}{[G_{12}(\lambda, y_{2i:m_2:n_2})]^{\theta+2}} \left(\frac{\partial G_{12}(\lambda, y_{2i:m_2:n_2})}{\partial \gamma} \right) \left(\frac{\partial G_{12}(\lambda, y_{2i:m_2:n_2})}{\partial \lambda} \right) + \psi_5(\lambda, y_{2i:m_2:n_2}).$$

and $\psi_1(\lambda^{j-1}, T_j)$ is the as $\psi_1(\lambda^{j-1}, y_{ji:m_j:n_j})$ with $y_{ji:m_j:n_j}$ replaced by T_j. By the same way for $\psi_2(\lambda^{j-1}, T_j), \psi_3(\lambda^{j-1}, T_j), \psi_4(\lambda^{j-1}, T_j), \psi_5(\lambda^{j-1}, T_j)$ and $\psi_6(\lambda^{j-1}, T_j)$.
